# INFERR-Iron infusion in haemodialysis study: INtravenous iron polymaltose for First Nations Australian patients with high FERRitin levels on haemodialysis—a protocol for a prospective open-label blinded endpoint randomised controlled trial

**DOI:** 10.1186/s13063-021-05854-w

**Published:** 2021-12-02

**Authors:** Sandawana William Majoni, Jane Nelson, Darren Germaine, Libby Hoppo, Stephanie Long, Shilpa Divakaran, Brandon Turner, Jessica Graham, Sajiv Cherian, Basant Pawar, Geetha Rathnayake, Bianca Heron, Louise Maple-Brown, Robert Batey, Peter Morris, Jane Davies, David ( Kiran) Fernandes, Madhivanan Sundaram, Asanga Abeyaratne, Yun Hui Sheryl Wong, Paul D. Lawton, Sean Taylor, Federica Barzi, Alan Cass, for the INFERR study Group

**Affiliations:** 1 Division of Wellbeing and Preventable Chronic Diseases, Menzies School of Health Research, Charles Darwin University, Darwin, Northern Territory Australia; 2 Department of Nephrology, Division of Medicine, Royal Darwin Hospital, P.O. Box 41326, Casuarina, Darwin, Northern Territory Australia; 3 Flinders University and Northern Territory Medical Program, Royal Darwin Hospital Campus, Darwin, Northern Territory Australia; 4 Department of Nephrology, Division of Medicine, Alice Springs Hospital, Alice Springs, Northern Territory Australia; 5 Chemical Pathology–Territory Pathology, Department of Health, Northern Territory Government, Darwin, Northern Territory Australia; 6 Department of Endocrinology, Division of Medicine, Royal Darwin Hospital, Darwin, Northern Territory Australia; 7 New South Wales Health, St Leonards, NSW Australia; 8 Child Health Division, Menzies School of Health Research, Charles Darwin University, Darwin, Northern Territory Australia; 9 Department of Pediatrics, Division of Women, Children and Youth, Royal Darwin Hospital, Darwin, Northern Territory Australia; 10 Department of Infectious Diseases, Division of Medicine, Royal Darwin Hospital, Darwin, Northern Territory Australia; 11 Global and Tropical Health Division, Menzies School of Health Research, Charles Darwin University, Darwin, Northern Territory Australia; 12 The Central Clinical School, Monash University & Alfred Health, Melbourne, Australia; 13 UQ Poche Centre for Indigenous Health, The University of Queensland, St Lucia, Queensland 4067 Australia

**Keywords:** Aboriginal and Torres Strait Islander Australians, First Nations Australians, Anaemia, Chronic kidney disease, Maintenance haemodialysis, Ferritin, Iron deficiency, Intravenous iron

## Abstract

**Background:**

The effectiveness of erythropoiesis-stimulating agents, which are the main stay of managing anaemia of chronic kidney disease (CKD), is largely dependent on adequate body iron stores. The iron stores are determined by the levels of serum ferritin concentration and transferrin saturation. These two surrogate markers of iron stores are used to guide iron replacement therapy. Most Aboriginal and/or Torres Islander Australians of the Northern Territory (herein respectfully referred to as First Nations Australians) with end-stage kidney disease have ferritin levels higher than current guideline recommendations for iron therapy. There is no clear evidence to guide safe and effective treatment with iron in these patients. We aim to assess the impact of intravenous iron treatment on all-cause death and hospitalisation with a principal diagnosis of all-cause infection in First Nations patients on haemodialysis with anaemia, high ferritin levels and low transferrin saturation

**Methods:**

In a prospective open-label blinded endpoint randomised controlled trial, a total of 576 participants on maintenance haemodialysis with high ferritin (> 700 μg/L and ≤ 2000 μg/L) and low transferrin saturation (< 40%) from all the 7 renal units across the Northern Territory of Australia will be randomised 1:1 to receive intravenous iron polymaltose 400 mg once monthly (200 mg during 2 consecutive haemodialysis sessions) (Arm A) or no IV iron treatment (standard treatment) (Arm B). Rescue therapy will be administered when the ferritin levels fall below 700 μg/L or when clinically indicated. The primary outcome will be the differences between the two study arms in the risk of hospitalisation with all-cause infection or death. An economic analysis and several secondary and tertiary outcomes analyses will also be performed.

**Discussion:**

The INFERR clinical trial will address significant uncertainty on the safety and efficacy of iron therapy in First Nations Australians with CKD with hyperferritinaemia and evidence of iron deficiency. This will hopefully lead to the development of evidence-based guidelines. It will also provide the opportunity to explore the causes of hyperferritinaemia in First Nations Australians from the Northern Territory.

**Trial registration:**

This trial is registered with The Australian New Zealand Clinical Trials Registry (ANZCTR): ACTRN12620000705987. Registered 29 June 2020.

**Supplementary Information:**

The online version contains supplementary material available at 10.1186/s13063-021-05854-w.

## Administrative information

Note: the numbers in curly brackets in this protocol refer to SPIRIT checklist item numbers. The order of the items has been modified to group similar items (see http://www.equator-network.org/reporting-guidelines/spirit-2013-statement-defining-standard-protocol-items-for-clinical-trials/).

**Table:** 

Title	INFERR-Iron Infusion in Haemodialysis Study: INtravenous Iron Polymaltose for First Nations Australian Patients with High FERRitin Levels on Haemodialysis: A Protocol for a Prospective Open-Label Blinded Endpoint Randomised Controlled Trial
Trial registration {2a and 2b}.	Trial registry name: The Australian New Zealand Clinical Trials Registry (ANZCTR) Trial identifier: ACTRN12620000705987
Protocol version {3}	27th August 2020; Version 2.0,
Funding {4}	The trial is funded by National Health and Medical Research Council (NHMRC) Project Grant #1163841
Author details {5a}	Sandawana William Majoni^1,2,5^, Jane Nelson^1^, Darren Germaine^1^, Libby Hoppo^1^, Stephanie Long^1^, Shilpa Divakaran^1,2^, Brandon Hoschke^1^. Jessica Graham^1,^ Jack Roe^1^, Sajiv Cherian^1,5,6^, Basant Pawar^6^, Geetha Rathnayake^5,7^, Bianca Heron^2^, Louise Maple-Brown^1,3^, Robert Batey^6,8^, Peter S Morris^9,10^, Jane Davies^4, 13^, David (Kiran) Fernandes^6^, Madhivanan Sundaram^2^, Asanga Abeyaratne^1,2,5^, Jaquelyne T Hughes^1^, Yun Hui Sheryl Wong^1,2^, Paul D Lawton^1,11,^ Sean Taylor^1,2^, Federica Barzi^1,12^, Alan Cass^1^ ^1^Wellbeing and Preventable Chronic Diseases, Menzies School of Health Research, Charles Darwin University, Northern Territory, Australia ^2^Department of Nephrology, Division of Medicine, Royal Darwin Hospital, Darwin, Northern Territory, Australia. ^3^Department of Endocrinology, Division of Medicine, Royal Darwin Hospital, Darwin, Northern Territory, Australia. ^4^Department of Infectious Diseases, Division of Medicine, Royal Darwin Hospital, Darwin, Northern Territory, Australia. ^5^Flinders University and Northern Territory Medical Program, Royal Darwin Hospital Campus, Darwin, Northern Territory, Australia. ^6^Department of Nephrology, Division of Medicine, Alice Springs Hospital, Alice Springs, Northern Territory, Australia. ^7^Chemical Pathology–Territory Pathology, Department of Health, Northern Territory Government, Northern Territory, Australia ^8^New South Wales Health, NSW ^9^Child Health Division, Menzies School of Health Research, Charles Darwin University, Northern Territory, Australia ^10^Department of Pediatrics, Division of Women, Children and Youth, Royal Darwin Hospital, Darwin, Northern Territory, Australia. ^11^The Central Clinical School, Monash University & Alfred Health ^12^ UQ Poche Centre for Indigenous Health, The University of Queensland, St Lucia Queensland 4067 ^13^Global and Wellbeing Division, Menzies School of Health Research, Charles Darwin University, Northern Territory, Australia
Name and contact information for the trial sponsor {5b}	The trial Sponsor is the Menzies School of Health Research, PO Box 41096 Casuarina, Northern Territory 0811. Contact details: Phone: + 61 8 8946 8600. Website: www.menzies. edu.au.
Role of sponsor {5c}	Menzies School of Health Research, as the administering organization for the NHMRC Grant for the INFERR study, is the trial sponsor. Menzies School of Health Research is responsible for financing, initiating, managing, developing, and coordinating the Study The study funders have no role or authority over study design, and will not have any role during collection, management, analysis, and interpretation of data; writing of the report; or the decision to submit the report for publication.

## Introduction

### Background and Rationale{6a}

Rates of dialysis-requiring end-stage kidney disease (ESKD) among Aboriginal and Torres Strait Islander Australians (herein respectfully referred to as First Nations Australians) of the Northern Territory (NT) are some of the highest in the world [1, 2]. Anaemia is a major complication of ESKD [3]. Prevalence rates of anaemia are also high among First Nations Australians [4–6]. Anaemia is associated with reduced quality of life, increased risk of cardiovascular disease, hospitalisations, cognitive impairment, and mortality [7, 8]. Therapy with erythropoiesis-stimulating agents (ESAs) is the main approach for correcting anaemia in people with chronic kidney disease (CKD) and work most effectively when the patient is replete in iron stores. The appropriate use of ESAs improves quality of life and reduces morbidity and mortality [3]. One of the major causes of anaemia in people with CKD is iron deficiency [9]. Iron deficiency is also the commonest cause of poor response to ESA therapy [10, 11].

The effective treatment of anaemia in maintenance haemodialysis (MHD) patients therefore requires both the accurate assessment of iron status, and the safe and appropriate correction of iron deficiency. Several published guidelines are available to guide iron replacement in patients with CKD [12–15] (Supplementary Table 1). Iron therapy using these guidelines is largely dependent on the levels of serum ferritin and transferrin saturation (TSAT) [the ratio of serum iron to the total iron-binding capacity (TIBC) as a percentage], both surrogate markers of iron status. Ferritin is an intracellular protein which stores excess iron, and low serum ferritin is a marker of iron deficiency. However, ferritin is also an acute phase protein, and increased serum ferritin concentration, along with increases in other acute phase reactants such as C-reactive protein (CRP), is observed in patients who have inflammation [6, 16]. There is also growing evidence that high serum ferritin concentration is independently associated with high rates of hospitalisations, cardiovascular disease and mortality [6, 17, 18].

True iron deficiency is indicated by the co-occurrence of both low ferritin and low TSAT levels. Functional iron deficiency is characterised by an impaired iron release from body stores [19]. This is thought to be due to reticuloendothelial cell iron blockade; serum ferritin is normal or high and serum TSAT levels are low. In patients with CKD, both true iron deficiency and functional iron deficiency need treatment with iron replacement therapy [14]. In First Nations Australians in the NT, anaemia in MHD patients co-exists with ESA-hyporesponsiveness, high serum ferritin concentrations and high serum CRP [16] in a population with heavy burden of cardiovascular, infectious diseases, hospitalisations and premature mortality. High serum ferritin levels have been recognised in this population for almost 20 years, and since these levels may be due to chronic inflammation [16, 20–23], there is significant uncertainty regarding the appropriate management of anaemia [16, 23] using serum ferritin levels as a marker of iron status. The recently replaced NT iron management protocol was developed to guide iron replacement in MHD patients with serum ferritin levels above 500μg/L associated with low TSAT (Supplementary Table 2). Evidence to guide safe and effective use of intravenous iron in this population is urgently required.

In a study of the effectiveness of this protocol in correcting iron deficiency in 197 NT patients on MHD, we found patients were poorly responsive to ESA treatment and received higher annual doses of iron treatment both in absolute terms and relative to the haemoglobin response [16]. In another cohort, high ferritin levels in First Nations MHD patients were only partly explained by iron sufficiency as indicated by high TSAT [23]. The retrospective design of these 2 studies meant that the possible presence of iron overload [24], which is a clear risk in this situation, could not be determined. Despite this, the higher iron doses observed [16] suggest iron overload is a potential harm. We thus identified the need for a prospective study to investigate the safe and effective administration of iron among First Nations patients on MHD with high ferritin levels [16, 23, 24].

Findings of studies that we conducted in the NT [6, 16, 23] and evidence from other international studies demonstrate a need to use serum ferritin levels and TSAT in conjunction with other markers of iron status, particularly in patients with a high serum ferritin concentration. A report from the United Kingdom National Institute for Health and Care Excellence (NICE) has discouraged the use of either measure alone due to significant problems with lack of accuracy in determining the correct treatment [14]. Other potential markers of iron status include hepcidin [25–27], soluble transferrin receptor [28] (shown to be a better marker of iron stores than ferritin in First Nations children) [29], low mean corpuscle volume (MCV) and hypochromic, microcytic RBCs on blood film, percentage hypochromic red cells (PHRC) and reticulocyte haemoglobin content [30].

One potential and unexplored cause of high ferritin is the association with the metabolic syndrome in our patients [31]. Metabolic syndrome and diabetes are highly prevalent conditions among First Nations ESKD patients, but the association with raised ferritin has not been investigated. Examining the association of ferritin with more specific inflammatory markers such as interleukin-6 (IL-6) and tumour necrosis factor-α (TNF-α) will help to confirm if the ferritin is due to causes other than inflammation [32, 33].

A recent study showed that serum ferritin (200–500 μg/L) correctly reflected liver iron stores, as assessed by magnetic resonance imaging (MRI) (FerriScan®), in haemodialysis patients without overt inflammation or malnutrition [34]. The authors concluded that current ferritin target values should be lowered to avoid iron overload. However, ferritin levels in their patients were much lower than in our First Nations MHD patients [34]. Another study has the opposite conclusion that transferrin saturation and serum ferritin levels were poor markers of liver and cardiac iron deposition in MHD subjects [35]. In patients with high ferritin levels receiving iron treatment, it is crucial to monitor iron overload by measuring liver iron non-invasively with MRI-FerriScan® [36, 37]. Monitoring liver fibrosis (using FibroScan®) and cirrhosis is also crucial [31, 38–40]. Considering the high prevalence of diabetes mellitus and metabolic syndrome in the First Nations Australian population, it may be hypothesised that a high prevalence of hepatic steatosis and fibrosis exists as encountered in dialysis patients more and more frequently in developed countries as well as the dysmetabolic iron overload [6]. There is a need to identify those patients with these underlying liver diseases, to adapt iron dosage and to monitor the safety of IV iron in this setting. This peculiar safety issue seems particularly relevant since indiscriminate IV iron has recently been shown to either trigger or aggravate non-alcoholic fatty liver disease / non-alcoholic steatohepatitis in dialysis patients suggesting a double-edged iron sword [6].

In some studies, iron treatment has been associated with adverse effects (infections, cardiovascular complications and hospitalisations) especially if doses of > 1000 mg are given over 6 months [41]. Our patients are already at high risk of infections such as high rates of melioidosis during the wet season and high prevalence cardiovascular disease [42, 43].

Current evidence of treatment in patients with high serum ferritin levels is from two North America clinical trials. The Dialysis Patients’ Response to IV Iron with Elevated Ferritin (DRIVE) study showed that administration of ferric gluconate (125 mg for eight treatments) was superior to no iron therapy in anaemic dialysis patients receiving adequate ESA dosages with a ferritin 500 to 1200 ng/ml and TSAT ≤ 25% [44]. In our patient population, only 5.6% of the First Nations patients would fit entry criteria for the DRIVE study [16].

The follow-up DRIVE II study was a 6-week observational extension of DRIVE which concluded that ferric gluconate maintained haemoglobin and allowed lower ESA doses [45]. These two studies partly informed the development of our recently replaced treatment protocol tailored to the high levels of ferritin in our patients (supplementary Table 1).

In The Proactive IV irOn Therapy in hemodiALysis patients (PIVOTAL) trial, incident (< 12 months) haemodialysis patients were randomised 1:1 to high-dose IV iron sucrose versus a reactive low-dose IV iron sucrose in haemodialysis patients across the UK [46–48]. Patients with ferritin levels > 700 μg/L were excluded. The trial confirmed the safety and efficacy of high-dose IV iron at a maximum dose of 400 mg of iron once a month [46].. The exclusion of patients with high ferritin makes the results of this trial not generalisable to our First Nations dialysis population since over 80% of the patients have a ferritin higher than 700 μg/L [16].

In a new protocol used by dialysis units across the NT for patients with ferritin < 700 μg/L and TSAT < 40%, a maximum dose of 400 mg of IV iron once monthly is convenient and provide the best current evidence based on the PIVOTAL trial for dosing IV iron in dialysis patients (see additional file 1).

The INFERR clinical trial assesses the efficacy of IV iron in First Nations MHD patients with high serum ferritin levels. We hypothesise that intravenous iron therapy will safely and effectively improve haemoglobin concentration and reduce the frequency of anaemia among First Nations Australians on MHD who have anaemia with low transferrin saturation (less than 40%) coexisting with high serum ferritin concentrations (> 700 μg/L and less ≤ 2000 μg/L).

### Objectives {7}

#### Primary objective

The primary objective is to assess the impact of intravenous iron treatment on all-cause death and hospitalisation with a principal diagnosis of all-cause infection in First Nations patients on MHD with anaemia, high ferritin levels and low transferrin saturation

#### Secondary objectives

The secondary objectives are as follows: (1) to document serious adverse events – major cardiovascular events, rates of infections and all-cause hospitalisation rates associated with the intravenous iron therapy [2]. To assess the relationship between ferritin, total body iron levels, measures of iron status, inflammation and liver disease [3]. To determine the costs that associate with intravenous iron treatment in this setting.

### Trial design {8}

**Fig. 1:**
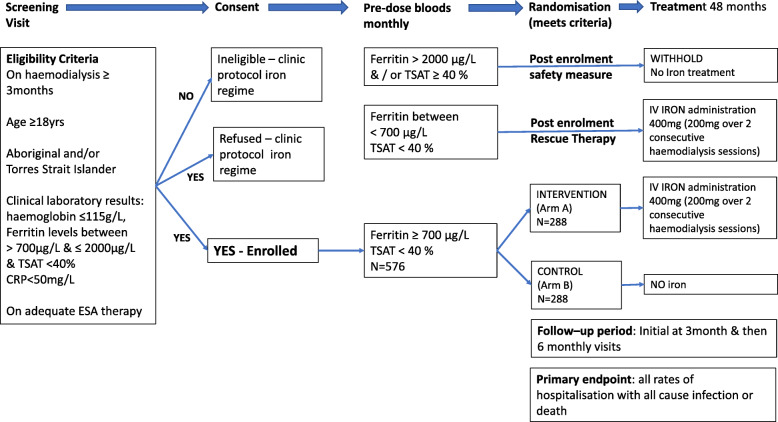
Overview of INFERR trial design

**Fig. 2:**
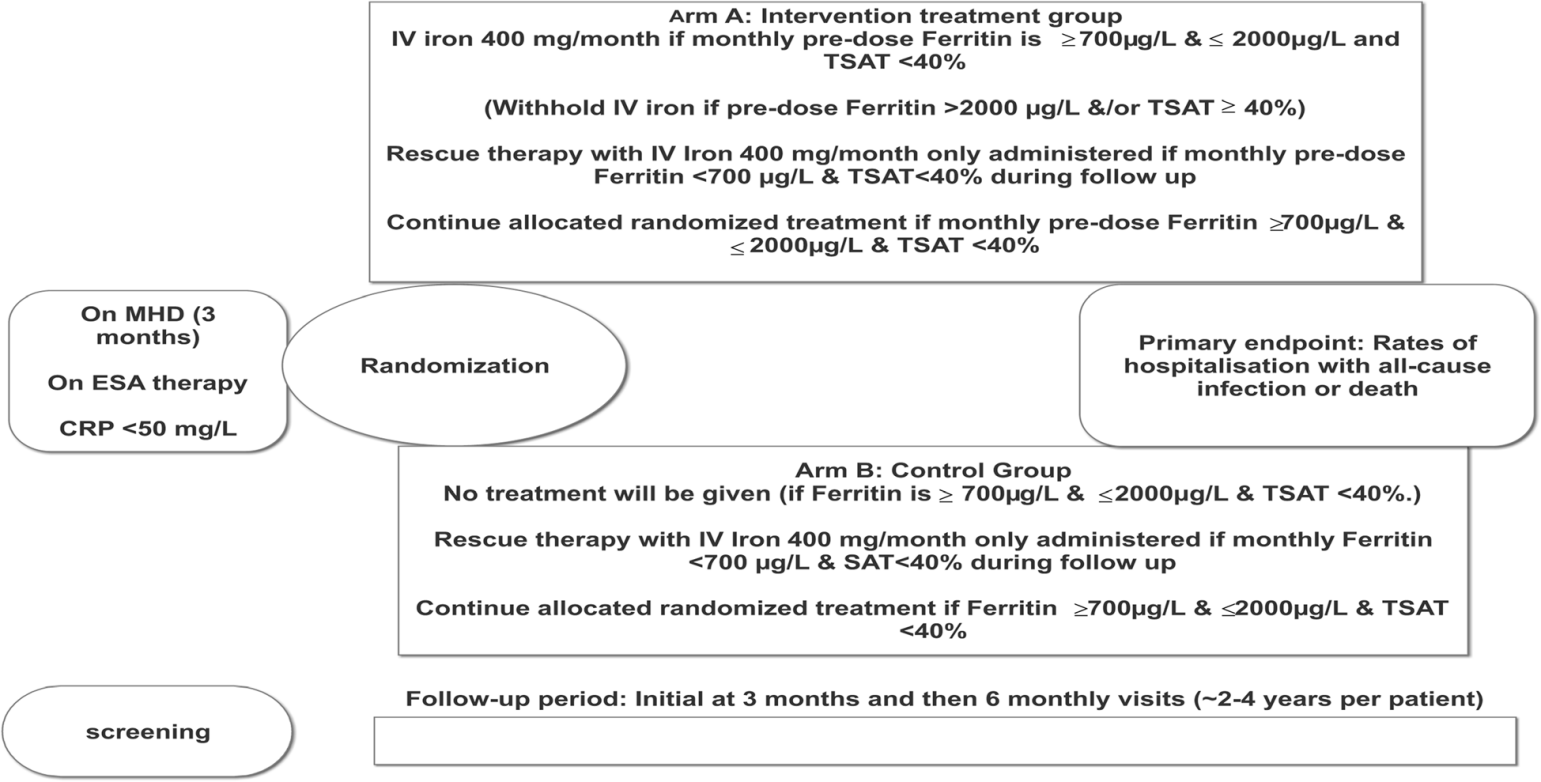
INFERR Clinical trial design and treatment allocation

INFERR is a multi-centre, parallel group, prospective open-label blinded endpoint randomised controlled superiority trial which compares intervention intravenous iron therapy and control group receiving standard care in adult First Nations Australians on MHD with a serum ferritin of over 700 μg/L (Figs. 1 and 2).

## Methods: participants, interventions and outcomes

### Study setting {9}

The study is conducted in all the nine renal haemodialysis units across the Northern Territory (NT) of Australia where over 85% of dialysis patients are First Nations Australians. First Nations Australians on MHD for over 3 months are recruited in the study. Participating units include the 7A Dialysis Unit at the Royal Darwin Hospital, Nightcliff and Palmerston renal dialysis units in Darwin, Katherine Dialysis unit, Tiwi Islands Dialysis unit, Tennant Creek Dialysis unit, the inpatient renal ward at Alice Springs Hospital and the Gap Road and Flynn Drive Dialysis units in Alice Springs.

### Eligibility criteria {10}

Participant inclusion criteria include the following: [1] male or female aged ≥ 18 years old [2]; identify as Aboriginal and/or Torres Strait Islander (First Nations) [3]; on maintenance haemodialysis for ≥ 3 months [4]; the following clinical laboratory results: (a) haemoglobin ≤ 115 g/L, (b) ferritin levels between ≥ 700 μg/L and ≤ 2000 μg/L, (c) TSAT < 40%, (d) CRP < 50 mg/L [5]; willing to join the study and can provide informed consent; and [6] on adequate erythropoietin-stimulating agent (ESA) therapy.

The following are the participant exclusion criteria: [1] History of known allergic or adverse or hypersensitivity reactions to iron polymaltose [2]; already receiving iron unless stopped for ≥ 4 weeks at the time of recruitment [3]; have received blood transfusion within the last 4 weeks [4]; known iron overload, haemochromatosis, haemoglobinopathy, haemolytic anaemia, aplastic anaemia, lymphoproliferative disease or cancer or on current cancer treatment [5]; participant’s primary clinician unwilling to enrol [6]; not planning to remain resident in the NT for at least 12 months [7]; ferritin > 2000 μg/L [8]; TSAT ≥ 40% [9]; CRP ≥ 50 mg/L [10]; life expectancy < 6 months based on the judgement of the investigator [11]; living-donor transplant scheduled within 12 months; and [12] Scheduled to switch to peritoneal dialysis.

Temporary exclusion criteria are as follows: The participant may not enter the trial at any given time if fever > 38 °C, or has evidence of active bacterial infection, or CRP ≥ 50 mg/L and current severe acute asthma, eczema and any allergies. These can be considered once these have normalised and are under control.

### Who will take informed consent {26a}

The consent process is carried out by the study team of trained research nurses delegated the responsibility by the principal investigator with the assistance of an Aboriginal research assistant. The research team explains the rationale and methodology of the study face to face to potential study participants from the dialysis patients and their families and carers. Patients and their families and carers are given the opportunity to ask questions and consider whether to participate in the trial. An informed consent discussion is held with each participant. The information for the discussion is provided in written and oral formats that have been approved by the Human Research Committees (HRECs) and in a language comprehensible to the potential participant, using interpreters where indicated.

The information presented details the exact nature of the trial and what is expected of the participant including any risks or benefits in taking part. It is clearly stated that the participant is free to withdraw from the trial at any time for any reason without prejudice to future care, and with no obligation to give the reason for withdrawal. The participants are allowed ample time to go through the Participant Information Sheet and ask questions.

The participant personally signs and dates the latest approved version of the consent form, as will the investigator or the delegated study team member (who conducted the consent discussion). If one was used, an interpreter also signs and dates the consent form. Where the participant is illiterate, they can sign the consent form with their mark rather than their signature. A witness must be present during the entire consenting process and must be able and willing to sign the consent form also. A copy of the Participant Information Sheet and consent form is provided to the participant. No trial-related procedure is undertaken before documented informed consent is obtained. The original copy of the consent form is retained in the Investigator Site File (ISF) at the recruitment site and a copy placed in the patient’s medical records. The person conducting the consent discussion will document this in the participant’s medical record and enter data into the Day 1 Case Report Form (CRF)

### Additional consent provisions for collection and use of participant data and biological specimens {26b}

Additional consent will be obtained for the FerriScans for the determination of liver iron, ultrasound scans of the liver and liver FibroScans for the determination of any liver disease.

## Interventions

### Explanation of choice of comparators {6b}

In the standard clinical protocol used by dialysis units across the NT for patients with ferritin < 700 μg/L and TSAT < 40%, a maximum dose of 400 mg of IV iron once monthly is used and is based on the best current evidence for iron administration in haemodialysis patients based on the results of the PIVOTAL trial for dosing IV iron in haemodialysis patients [46] (see additional file 1). Using this protocol, participants in the study will receive either IV iron 400 mg every month (200 mg during 2 consecutive haemodialysis sessions) or no IV iron as the comparator.

### Intervention description {11a}

#### Treatment of study participants

Participants are randomised to either Arm A: Intervention IV Iron treatment group (Figs. 1 and 2), or Arm B: Control group receiving no intravenous iron treatment (Figs. 1 and 2). After the randomisation, participants receive monthly treatment as per their randomisation of either monthly intravenous iron if serum ferritin ≥ 700 μg/L and ≤ 2000 μg/L and TSAT < 40% (Arm A) or no IV iron treatment (Arm B) for up to 36 months. Study treatment is withheld if monthly testing demonstrated ferritin > 2000 μg/L and/or TSAT ≥ 40%. These markers will be measured prior to administration of Arm A or B treatment. For both Arms A and B, if the serum ferritin level is < 700 μg/L and TSAT < 40%, rescue therapy is administered. All treatment is administered during dialysis via the ports on the dialysis circuits (see additional file 2).

#### Intervention treatment Arm A and the control Arm B

Intravenous iron polymaltose is administered according to the standard clinical protocol during haemodialysis. For Arm A, patients receive IV iron polymaltose 200 mg during each of the first 2 haemodialysis sessions at the start of the study, and during each of the first 2 dialysis sessions of the week following the monthly blood tests for all subsequent months (i.e. 400 mg per month). If monthly testing demonstrates ferritin > 2000 μg/L and/or TSAT ≥ 40%, IV iron is withheld for the month. The IV iron is restarted as per protocol when the monthly tests demonstrate ferritin > 700 μg/L and ≤ 2000 μg/L and TSAT < 40%. Rescue therapy of 400 mg of iron polymaltose is administered intravenously on haemodialysis if ferritin ≤ 700 μg/L and TSAT < 40% or as indicated by the treating nephrologist (see below). If monthly testing demonstrates ferritin > 700 μg/L and ≤ 2000 and TSAT < 40%, patients will continue with their allocated randomised treatment (Fig. 2).

### Criteria for discontinuing or modifying allocated interventions {11b}

#### Rescue therapy

For both Arms A and B, if monthly blood tests demonstrate ferritin **<** 700 μg/L and TSAT **<** 40%, 200 mg of intravenous iron polymaltose is administered during each of the first two haemodialysis sessions in the second week after the monthly blood tests. When the monthly blood tests indicate ferritin **>** 700 μg/L and ≤ 2000 μg/L and TSAT **<** 40%, the rescue therapy will be ceased, and participants continue to receive their allocated study treatment.

#### Withholding of therapy

For both arms, if monthly blood tests demonstrate ferritin ***>*** 2000 μg/L and/or TSAT ≥ 40%, study treatment is withheld. When the monthly blood tests indicate ferritin ≤ 2000 μg per litre and TSAT ***<*** 40%, patients continue to receive their allocated study treatment.

### Strategies to improve adherence to interventions {11c}:

#### Training of site nurses and nephrologists

All site staff at each participating dialysis unit are trained in the study protocol, Standard Operating Procedures (SOPs) and their reporting requirements by the Clinical Trial Manager or trained research nurse, a study chief investigator or delegate, prior to the site being opened for recruitment. All site investigators complete a computer-based training course in Good Clinical Practice and assist in providing robust training to their respective site staff.

The Clinical Trial Manager or delegate have regular phone, email or video conferencing contact with each participating dialysis unit.

### Relevant concomitant care that are permitted or prohibited during the trial {11d}

#### ESA therapy

All patients should be on adequate ESA dose at the time of randomisation. The ESA dose is determined according to the routine ESA dosing protocol. ESA dosing can be adjusted by the caring clinical teams as per the usual ESA administration protocol***.***

#### Documentation in patient’s medical record

A sticker is placed in the patient’s dialysis medical record (one on the progress notes on the day of randomisation, and one in the front inside cover of the dialysis record and on the medical record [“old notes”] if one exists). This sticker will alert clinicians that the patient has been randomised to the INFERR study, with a brief explanation of the study, and confirmation that the participant has provided written informed consent.

A copy of the study synopsis is also placed in the medical notes of the patient. A checklist of study procedures is also placed in the notes.

For electronic medical records, an electronic “sticker” is used, and appropriate annotations are made in the Primary Care Information System (PCIS) as well as letters in the Clinical Workstation (CWS). This is routine practice for all patients within the NT renal services.

#### Checking of medical records

The medical records (paper or electronic) are checked 3 months post randomisation (± 1 month) and at least 3 monthly (± 2 months) thereafter for up to 36 months by the study team to ensure adherence to the study protocol.

### Provision for post-trial care {30}

Participants found to have evidence of liver disease on ultrasound scans and/or FibroScans and those found to have iron overload on FerriScans will be referred to the liver clinics and haematologists respectively. All types of adverse events that occur from the day of randomisation to the end of follow-up will be reported to the sponsor and the participants’ usual clinical teams as soon as possible after the research staff identify the event. Participants will be given IV iron as per standard IV iron administration protocol post-trial as needed.

### Study outcomes {12}

#### The primary outcome

This will be involving an intention-to-treat analysis of the differences between the two study arms in the risk of hospitalisation with all-cause infection or death assessed in a time-to-first event analysis.

The primary outcome will be measured from the time of randomi**s**ation every **3** months to the end of the follow-up period.

#### Secondary outcomes

1. Differences between the 2 treatment groups in change from baseline of haemoglobin (Hb) level at 3, 6, 12, 24, 30 and 36 months and over the follow-up period.
2. The differences between the two study treatment arms assessed in a time-to-first-event analysis of the following, all are measured every 3 months after randomised unless otherwise stated:
  - A composite of non-fatal myocardial infarction, non-fatal stroke, hospitalisation for heart failure or death from any cause
  - All-cause death
  - Hospitalisation with any new diagnosis of infections
  - Hospitalisation with a principal diagnosis of infection
  - Vascular access thrombosis
  - Peripheral vascular disease and/or amputations
  - All-cause hospitalisation
  - Fatal and non-fatal cardiovascular event (myocardial infarctions (MI), stroke and hospitalisation for heart failure (HF))
  - Fatal or non-fatal MI
  - Fatal or non-fatal stroke
  - Heart failure hospitalisation
  - Length of hospital stay
  - ESA resistive Index (ERI), defined as average weekly erythropoietin (EPO) dose per kg body weight (wt) per average haemoglobin (Hgb), over a 3-month period (ERI = (EPO/wt)/Hgb))
3. Serious adverse events
  - All serious adverse events
  - All hospital admissions
  - Infections associated with hospitalisation
  - All-cause mortality
  - Iron overload (measured by magnetic resonance imaging (MRI) FerriScan^@^ of the liver) measured within 6 months post randomisation and at between 12 and 24 months post randomisation
4. Cost analysis: an analysis of the financial costs of the treatment will be performed assessing the costs of ESA, iron, hospital admissions and procedures for the duration of follow-up among all patients assigned to intravenous iron compared to no iron treatment.

#### Other outcomes assessed with non-randomized analyses

1. Assessment of *factors associated with high serum ferritin levels*: Association between ferritin and (a) other measures of iron status (serum hepcidin, transferrin saturation (TSAT), soluble transferrin receptor, percentage hypochromic red cells, reticulocyte haemoglobin content, liver iron), (b) Inflammatory markers (C-reactive protein (CRP), tumour necrosis factor alpha, interleukin 1 (IL1) and interleukin 6 (IL6)) and others (infection rates, CV events, hepatitis, medications, comorbidities, dialysis vintage, dialysis access, hospitalisations, time variation of ferritin)
2. *Assessment of liver disease:* liver function tests *(*LFTs), hepatitis B status, ultrasound and FibroScan of the liver
3. *Assessment of the comparability of the current ferritin assay platforms*: the Abbott ARCHITECT versus the Vitros® Ortho Clinical Diagnostics (OCD) platforms
4. *Analysis of defined primary and secondary outcomes*: by average weekly dialysis attendance (according to both ordinal attendance group and using attendance as a continuous variable)
5. *Analysis of defined primary and secondary outcomes*: by markers of inflammation

#### Tertiary outcomes: comparison between the two treatment groups of the following all measured at every 3 months

1. Cumulative dose of iron
2. Serum ferritin levels
3. TSAT
4. Platelet count
5. Serum albumin levels

### Participant timeline {13}

The participant timelines and scheduled visits are shown in Fig. 2 and Tables 1 and 2.

**Table 1:** Schedule of visits, data collection and follow-up

Visit day	Pre-screen	Day 1 baseline	3 M	6 M	9 M	12 M	15 M	18 M	21 M	24 M	27 M	30 M	33 M	36 M
Visit Window			± 2 weeks	± 2 weeks	± 2 weeks	± 2 weeks	± 2 weeks	± 2 weeks	± 2 weeks	± 2 weeks	± 2 weeks	± 2 weeks	± 2 weeks	± 2 weeks
Check eligibility	x	x												
Informed consent		x												
Demographic data		x												
Medical history		x												
Dialysis history		x												
Sociodemographic history		x												
Randomise		x												
Blood test results^1^	x	x	x	x	x	x	x	x	x	x	x	x	x	x
Additional iron studies pathology^2, 3^		x	x							x				
MRI scan (FerriScan)^3^				x						x				
Ultrasound/FibroScan of the liver ^4^				x						x				
Clinical outcomes data collection			x	x	x	x	x	x	x	x	x	x	x	x
Vital status (alive)		x	x	x	x	x	x	x	x	x	x	x	x	x
Procedures		x	x	x	x	x	x	x	x	x	x	x	x	x
Hospitalisations		x	x	x	x	x	x	x	x	x	x	x	x	x
Concomitant medication		x	x	x	x	x	x	x	x	x	x	x	x	x
Adverse events		x	x	x	x	x	x	x	x	x	x	x	x	x

^1^ Monthly routine blood test results will be reviewed at all scheduled study time points

^2^FBC, Retics, CYTOK, ADIPO, STFR and HPCD

^3^MRI (FerriScan) will occur within 6 months of randomisation and again between 12 and 18 months from day 1 on approx. 100 willing participants

^4^Ultrasound (FibroScan) will occur within 6 months of randomisation and again between 12 and 18 months from day 1

^5^A comparison of ferritin assays will be performed at 1 month post randomisation on a small number of participants

**Table 2:** Study timelines

	2019				2020				2021				2022				2023			
Quarter	1	2	3	4	1	2	3	4	1	2	3	4	1	2	3	4	1	2	3	4
Finalisation of protocol																				
Development of eCRFs																				
Ethics applications																				
Protocol Amendment 1																				
Site preparation																				
Recruitment																				
Participants follow-up																				
Data cleaning and analysis																				
Writing of paper(s)																				

This project will run until the end of 2023, with a predicted recruitment period of 2.5 years (see Table 2).

### Sample size {14}

Maximal planned follow-up in the study will be 36 months (3 years). The 2-year rate of admission with first episode of infection or death in our patients on maintenance haemodialysis from 2000 to 2014 was 60%. In order to achieve a study power > 80% at 5% significance level in detecting a 30% reduction (from 60 to 48%) in the rate of first hospitalisation with all-cause infection or death, using the log-rank test (time-to-event modelling) assuming a 5% drop-out rate, 576 participants will be randomised 1:1 into each arm. The study also has > 90% power at 5% significance level of detecting a difference in haemoglobin of 5 g/L and standard deviation of 11.3 g/L based on published data [44, 45]. The sample size also provides enough power to determine differences within the ferritin strata.

### Recruitment {15}

#### Screening

All patients receiving maintenance haemodialysis in renal units across the Northern Territory undergo a series of clinical laboratory tests (haematology and serum chemistry) at the commencement of the month as part of routine care. These results are used to screen potential participants for eligibility into the study.

The treating renal team is approached for a discussion on potential recruitment of the patients into the trial, and for permission to review the patient’s medical notes. The study team assesses the eligibility criteria and completes the Pre-Screening Case Report Form (CRF). If ineligible, the patient is not approached. If considered eligible, the patient is approached for an informed consent discussion using the teach back method to determine if an interpreter is required to obtain consent. After consent is obtained, eligibility will be re-established prior to randomisation. Each unit maintains a log of all pre-screened patients along with their eligibility and consent outcomes in the medical records or notes. However, should a potential participant consent meet the temporary exclusion criteria at time of screening they will be re-approached for inclusion into the trial at a subsequent clinic visit.

To maximise recruitment, the research team spends a considerable time in the renal units providing information and education to potential participants using the teach back method. There is also a potential for expanding the recruitment to other renal units across Northern Australia to achieve the desired sample size.

## Assignments of interventions: allocations

### Sequence generation {16a}

Prior to proceeding with randomisation, the research nurse and/or investigator ensures informed consent has been obtained and documented and confirm that the participant is eligible to be enrolled. Participants are randomised using a module in the web-based study database, the Research Electronic Data Capture (REDCap) that is password protected and stored on a secure drive at Menzies School of Health Research. Compulsory fields required prior to randomisation are screening number (Study Identification number), confirmation of eligibility, age, hospital registration number (HRN), confirmation of consent, recruitment site (unit) and ferritin strata. Randomisation will be stratified by location (Top End and Central Australia) and ferritin strata (≤ 1200 μg/L and > 1200 μg/L) and will be in permuted blocks of variable block size. Participants are randomised in a 1:1 ratio to either Arm A: Intervention treatment group or Arm B: control group via REDCap, available 24 h per day, 7 days per week (Menzies School of Heath Research, Darwin).

### Concealment mechanism {16b}

The randomised sequence allocation is stored on the password-protected secure server by the trial statistician and will not be available to any investigators or member of study staff.

### Implementation {16c}

Randomisation is performed at the site with allocation occurring via the web-based database (REDCap), this only occurs after informed consent is obtained and eligibility confirmed. Participants are enrolled by Investigators or research nurses that have been delegated this responsibility by the principal or chief investigator (CI). To randomise the participant, the research nurse or investigator logs onto REDCap and enters the details required to obtain the treatment randomisation number assigned to the participant for that site.

## Assignment of interventions: blinding

### Who is blinded {17a}

This is an open-label blinded end point assessment trial. The intravenous iron treatments are supplied by a central pharmacy, either at the Royal Darwin Hospital or the Alice Springs Hospital and is prepared and administered by the nursing staff as per routine clinical care. The patient, nurses administering the drugs and investigators will not be blinded to the treatment allocation. The administration will be direct into the dialysis circuit as per the standard intravenous iron administration procedure on haemodialysis.

The investigators assessing and analysing the primary outcomes (see below—outcomes assessment committee) are blinded to treatment allocation. Independent outcomes Assessors not involved in any aspect of the study will review and complete a data collection form of all randomised study participants to independently determine the study outcomes (from both electronic and written medical records). In view of the relationships likely to develop between the research staff and some of the participants, it is essential that we engage independent outcomes assessors. The outcome assessment data collection forms and final assessment will be verified by two of the chief investigators.

### Procedures for unblinding outcomes if needed {17b}

This is an open-label blinded end point assessment trial. However, an Independent Data Safety and Monitoring Board (DSMB) reviews safety data, including serious adverse events and the primary endpoint events, and will advise on continuation of the study, or whether the study should be stopped prematurely because of safety concerns. In a closed session, the DSMB will be provided with descriptive analyses of study data by study labels “A” and “B” for the allocated treatment arms.

## Data collection and management

### Plans for assessment and collection of outcomes {18a}

#### Demographic and clinical data

A full and relevant clinical and social history will be obtained from each participant. Key factors of direct interest for this study include time on dialysis, dialysis access and dialysis access interventions, ideal dry weight, height, body mass index, waist hip ratio, primary renal diagnosis, comorbidities (liver, lung, diabetes, cardiovascular, etc.), current smoking status, history of current or previous alcohol intake, dietary history, any significant illnesses, previous Hb levels, a full medication list and history, and sociodemographic data.

#### Major events

Hospitalisation due to all-cause infections or death are documented as the primary outcome. All admissions overnight or longer are followed up with collection of detailed data on the admission. Data on all major vascular events, all dialysis access-related events including infections and vascular interventions not requiring hospital admission are also collected.

#### Data collection from routine care: routine blood tests

Haemodialysis patients have monthly blood tests as part of their routine care. Tests obtained as part of this routine screen that are analysed as outcomes and covariates of interest for this study include: Hb, CRP, full blood count (will include MCV and MCHC), liver function tests, iron studies (ferritin and TSAT), measures of dialysis adequacy (Urea reduction ratio and KT/V (a number calculated from change in urea pre- and post-dialysis, time on dialysis and volume of distribution of urea used to quantify dialysis adequacy). Every 3 months the following are also routinely collected: parathyroid hormone levels and glycated haemoglobin (HbA1c).

#### Additional blood tests

*Samples are collected at baseline and at 12 months for* (1) other measures of iron status (serum hepcidin levels, soluble transferrin factor levels, percentage hypochromic red cells, reticulocyte haemoglobin content) [2]; other causes of anaemia (thyroid function tests, vitamin B12 and folate (only at baseline) [3]; inflammatory markers (serum tumour necrosis factor (TNF) alpha, interleukin-1 (IL-1) and interleukin-6 (IL-6); and [4] and marker of the metabolic syndrome (serum total adiponectin).

#### Assessment of the comparability of the two ferritin concentration measurement methods

*From samples collected at around 1 month comparison of the* Abbott ARCHITECT i1000 (Abbot Diagnostics, USA) versus the Vitros Ortho® XT7600 in a sample of about 150 participants covering the analytical range and above of serum ferritin levels. Ferritin concentrations in the NT have previously been determined using the Abbott ARCHITECT which is the platform used by most laboratories in Australia. Territory Laboratories have recently changed to and now routinely use the Vitros Ortho Clinical Diagnostics platform for analyses of all biochemical analytes. The Vitros Ortho XT7600 gives ferritin concentrations results up to 40% lower than the Abbott ARCHITECT platform.

#### Determination of iron overload

In addition to serum ferritin and TSAT levels, liver iron levels will be determined by use of the non-invasive but accurate method, the magnetic resonance imaging (MRI)-based technique spin-density projection-assisted (SDPA) R2- MRI (FerriScan®) at baseline and at 12 months.

#### Other data from routine care

All patients are routinely followed up by their nephrologist every 3 months, and data from these follow-up appointments is reviewed.

#### Additional follow-up for the study

All participants have medical reviews by the study team at 3, 6, 9, 12, 15, 18, 21, 24, 27, 30, 33 and 36 months (primary outcome assessment).

#### Blinded endpoint assessment and adjudication

The composite primary endpoint is assessed by an independent blinded endpoint adjudication committee. This committee consists of three nephrologists appointed by the trial management committee. This committee is provided with an extract of study data that does not contain patient identifiers and does not contain any mention of treatment allocation or any detail about treatment of any kind, but does contain data relating to adverse events, procedures and concomitant medication changes linked to the primary and secondary outcomes of the study:

1. Demographic details (such as age and gender)
2. Comorbidities
3. Clinical details
4. Date and result of all blood results
5. Date and result of all other available clinical information
6. Hospitalisations and/or deaths

The members of the committee may request more information if needed, but this will only be provided if it is available and does not provide direct or indirect evidence of treatment allocation. Each of the three members of the committee will then independently determine if, in their view, the patient has met the primary endpoint. If there is a discrepancy between the three assessments, the majority will determine the endpoint.

### Plans to promote participant retention and follow-up {18b}

The study team makes regular contact (either by phone or preferably in person on the units) with the participants’ treating teams at 3 months and every 6 months. The purpose of this contact is to check compliance with the protocol in terms of study drug prescribing and ordering of routine clinical blood tests. Data is collected and entered into the corresponding visit CRF. Standard operating procedures (SOPs) will contain step by step details on how to recruit patients, collect data and monitor progress.

Participants have the right to choose to withdraw from the study at any time and the investigator may discontinue a participant from the study or from treatment if deemed appropriate at any time. This will have no impact on their normal dialysis and other treatment or care. Reasons why a participant may be withdrawn from the study include, but are not limited to, participant request, primary treating clinician’s request, participant was enrolled and is ineligible (either arising during the study or was overlooked at time of screening and enrolment). Participants will not be withdrawn due to adverse events. The decision to withdraw a participant from the study must be discussed with the coordinating investigators.

If the participant withdraws consent from participating in the study and also withdraws consent for collection of future information, no further evaluations will be performed, and no additional data will be collected. The sponsors may retain and continue to use any data or samples collected before such withdrawal of consent. Participants that are lost to follow-up will continue to be followed by collecting as much data as possible, if possible, until the end of the trial to avoid missing data. Participants withdrawn from the treatment by the treating clinicians will continue to be followed up to the end of the trial to avoid missing data and will be used in the intention-to-treat analysis. This study has allowed for 5% of participant drop-out. Withdrawn participants will not be replaced.

If a participant is withdrawn, the reason is recorded in REDCap.

### Data management {19}

Source documents are those where data are first recorded, and from which participants’ CRF data are obtained. These include but are not limited to hospital records both electronic and paper (which will include medical history, previous and current medications, any relevant radiography test, blood test results, haemodynamic parameters, and medical correspondence) and paper or electronic clinic records (which include vital status, recent medical history and relevant blood culture results). A further potential data source will be through telephone conversations with the study participant or their primary doctor or general practitioner.

Storage and archiving of study documents (CRFs and consent forms) are the responsibility of the site principal investigator and these will remain at the site of recruitment.

Data for this study is recorded via a secure, Electronic Data Capture (EDC) web-based system (REDCap). It is transcribed by the study team from the paper CRFs into REDCap. Data is stored in a re-identifiable manner in the database, using the unique screening number for each patient. The database contains validation ranges for each variable to minimise the chance of data entry errors. An audit trail will maintain a record of initial entries and changes made; reasons for change; time and date of entry; and username of the person who made the change. Data queries are raised by the Clinical Trial Manager and study monitor, and missing data or suspected errors are raised as data queries and resolved prior to database lock and analysis. The database contains in-line capability so that these queries and answers are logged as part of the audit trail.

For each potential participant screened (including screen failures), the screening eCRF is completed by the study team. For each participant enrolled, eCRFs must be completed. This also applies to records for those patients who fail to complete the study. The study team should ensure the accuracy, completeness and timeliness of the data reported in the eCRFs and in all required reports. A comprehensive validation check programme verifies the data and automatically generates discrepancies for resolution by the study team. Manual discrepancies can also be raised if necessary.

In addition, for selected data fields, accurate and reliable data collection is assured by verification of the eCRFs against the study team’s records by the study monitor (source document verification), and the maintenance of medication compliance is captured in the CRFs from the participant’s medication chart (source document) by the study team.

### Confidentiality {27}

REDCap is 21 CRF Part 11 capable. Currently, REDCap installations support electronic signatures by positively identifying the user through a unique username and password combination. All study participants are allocated a unique number at time of screening (screening number or study ID number), and this screening number is added to all the CRFs for that participant. The participants also have their hospital record number (HRN) recorded on the CRFs as this information is required to ensure the correct medical record is accessed during medical record reviews.

Access to the REDCap database will be given to the study personnel only, including the biostatistician and data management personnel. Data being analysed will be exported in de-identified format. As part of the data dictionary development process, individual fields are denoted as “identifiers.” When exporting a de-identified dataset, these variables are omitted. Identities of participants will not be revealed in the presentation or publication of any result from this study. All study personnel are educated about the importance of strictly protecting participants’ rights to confidentiality and can only access the data necessary for job completion. Participants will be informed of law-mandated instances in which confidentially could be breached.

The trial management committee will be the custodians of the final trial dataset. No-one outside the trial management committee will be given access to the data without the permission of the trial management committee. No identifying data will be given to any third parties at any stage. Following study close out and locking of the database, it will be stored on the servers of the Menzies School of Health Research.

### Plans for collection, laboratory evaluation, and storage of biological specimens for genetic or molecular analysis in this trial/future use {33}

#### Laboratory procedures

All blood tests are processed as per the local laboratory’s usual procedures. Some samples will need to be processed by the Menzies laboratories and Western Diagnostics as per standard clinical practice. Please refer to the INFERR Laboratory Standard Operating Procedure (SOP) for further information (Additional file 3).

#### Storage and testing of biological specimens

To ensure availability of relevant samples for study procedures, the laboratory linked to each site will freeze and store some samples. These will later be transported for archiving in – 80 °C freezers at the Menzies School of Health Research laboratory and Territory Pathology laboratories at Darwin and Alice Springs. Samples are transported to Menzies in batches at four specified time points in the study—3 months after the recruitment of participant # 100, 200, 300, 400 and 576. Samples are identified by their INFERR study number and local laboratory specimen ID number.

No biological specimens will be collected in the study for genetic or molecular analysis.

## Statistical methods

### Statistical methods for primary and secondary outcomes {20a}

#### Statistical analysis plan

Data will be reported in accordance with the Consolidated Standards of Reporting Trials (CONSORT) guidelines for reporting of randomised trials. Demographic, clinical, biochemical and outcome data will be described by allocation arm. Continuous normally distributed data will be summarised with mean and standard deviations (SD), continuous not normally distributed variables with median and inter-quartile ranges (IQR). Categorical variables will be described with frequency and corresponding percentage.

#### Analysing primary and secondary outcomes

Primary and secondary endpoints will be analysed according to the intention-to-treat principles (All participants data will be analysed according to the treatment allocation, regardless of what treatment they received and either they completed the follow-up or not).

The dichotomous primary outcome and all the dichotomous secondary events will be analysed using time-to-first-event regression using Cox proportional hazard models where the duration of the follow-up will be censored at the first between: date of outcome of interest, end of study, date of withdrawal (including withdrawal due to change in renal replacement therapy modality) or lost to follow-up. Given that these are competing events, sensitivity analysis will include analysis using Fine and Gray regression models [49]. The effect of the intervention will be estimated as compared to placebo using hazard ratios with corresponding 95% confidence intervals and *p* values. Recurrent event outcomes (i.e. rates of hospitalisation) will be analysed using the method of Ghosh and Lin [50] or, as an alternative, using random effects log-binomial models or one of the extensions of the Cox proportional hazards model [51, 52]. The approach choice will be guided by the distribution of the time to event and results of goodness of fit statistics.

Continuous outcomes assessed at multiple time points (e.g. haemoglobin) will be analysed using linear mixed models with a fixed effect for study allocation arm and time point and a random intercept to model the correlation of measurement taken within the same individual.

All the models will be univariable and include treatment arm only as independent variable (linear mixed models will also include a variable for time each outcome repeat was recorded).

Sensitivity analyses using a multivariable model to adjust for potential confounders will be performed when these factors appear to be not equally distributed between the two allocation arms.

*Pre-defined secondary stratified analyses* will be performed, comparing the effect of treatment between allocation arms according to:

(i) The presence of active hepatitis B carriage (defined dichotomously by the presence of either measurable hepatitis B DNA according to PCR by the ABBOTT REALTIME HBV assay, or Hep B surface antigen positivity measured according to immunometric immunoassay (serum), at any time during the study).
(ii) Average weekly haemodialysis attendance over the time included in the study, defined as an ordinal variable [in three groups: < 2.0 sessions/week, 2.0–2.75 sessions/week, > 2.75 sessions/week] for stratified analysis, as well as incorporated in Cox regression models with an interaction term between attendance group and treatment allocation group, as well as separately as an additional continuous variable in Cox proportional hazards regression modelling.
(iii) Baseline ferritin threshold (≤ 1200 μg/L and > 1200 μg/L)
(iv) According to presence/absence of comorbidities (diabetes, cardiovascular and cerebrovascular disease, lung disease).

### Interim analyses {21b}

The DSMB will conduct an interim analysis after 288 patients have been randomised (i.e. at 50% recruitment) and followed for 24 months (2 years) following the date of the first patient randomised.

The interim analysis will review outcome data and answer the following questions:

1. Are there any significant safety issues that may present an ethical issue in continuing the study? This may include adverse events, but also study conduct and protocol violations.
2. Is there overwhelming data suggesting the superiority of one arm that may present an ethical issue in continuing the study?

- Using the Haybittle-Peto rule, and the differences according to treatment allocation in risk of hospitalisation with all-cause infection or death as the outcome of interest, the study will be stopped early if there are differences according to treatment allocation in risk of hospitalisation with all-cause infection or death rate with a *p* value of less than or equal to 0.001.

3. Are there any other factors that may impact on the feasibility / usefulness of the study? For example, rate of enrolment, unexpected low rate of outcomes, unable to fund and protocol violations.

### Methods for any additional analysis (e.g. subgroup analyses) {20b}

#### Factors associated with high ferritin

Associations at baseline will be explored using both ferritin as a continuous outcome and as a categorical dichotomous outcome (high and not high) using multivariable linear and log-binomial regression models respectively. Factors considered for associations with the outcome are serum hepcidin, TSAT, soluble transferrin receptor, percentage hypochromic red cells, reticulocyte haemoglobin content, liver iron, CRP, tumour necrosis factor alpha, IL1, IL6, infection rates, CV events, hepatitis, medications, comorbidities, dialysis vintage, dialysis access, hospitalisations and time variation of ferritin. Only variables significant at a nominal level *α* = 0.1 in univariable models will be included in a multivariable model. Variables will then be excluded from the multivariable models according to a stepwise backward selection method and a final multivariable model identified that include only variables with a *p* < 0.1. Variables in the multivariable models will be considered to have an independent significant statistical association with the outcome if their *p* value is < 0.01, variables with a *p* value between 0.1 and 0.01 will be considered border-line significant/show a weak signal of association with the outcome.

#### Assessment of the comparability of the current ferritin assay platforms

Ferritin values assayed using the Abbott ARCHITECT and the Vitros® ortho XT7600 platforms will be compared using a one sample paired *t*-test. Bland-Altman plots and Passing and Bablok regression for comparing two methods will be used to assess agreement, constant disagreement or proportional disagreement of results obtained using the two platforms.

*Health economic analysis* will also be carried out, using the primary outcome measures for the trial to inform a modelling study. We will borrow cost and quality of life estimates from other studies/data sources.

### Methods in analysis to handle protocol non-adherence and any statistical methods to handle missing data {20c}

Both primary and secondary endpoints will be analysed according to the intention-to-treat principles (All participants data will be analysed according to the treatment allocation, regardless of what treatment they received and either they completed the follow-up or not). A secondary per-protocol analysis of all endpoints will be conducted. The per-protocol population will be all those who completed the treatment as allocated in the 2 groups.

Missing data will not be imputed. However, those with missing data will be described. This number is expected to be small (< 5% of randomised patients) and the same in each arm as maintenance haemodialysis patients attend haemodialysis three times per week, almost no First Nations dialysis patients leave the Northern Territory, and we will follow patients who move back to their community.

### Plans to give access to the full protocol, participant-level data, and statistical code {31c}

This protocol paper provides the full study protocol. Readers can contact the authors if they are interested in any other data or documentation of the study.

## Oversight and monitoring

### Composition of the coordinating centre and trial steering committee {5d}

#### Trial coordination teams

This trial is coordinated from the Menzies School of Health Research in Darwin, but a coordination team is also set up in Alice Springs. The teams coordinate the day to day running of the trial and meet weekly and work in collaboration with all the other investigators and research staff.

In both Alice springs and in Darwin, the team consist of:

- Clinical Trial manger and the Operational research support manager
- The principal investigators of the two centres
- Two other chief investigators of the study
- All the research nurses and Aboriginal research assistants
- Other members of the research investigator teams are invited to the meetings when needed

#### Trial management committee (TMC)

The trial management committee includes a chair, co-chair, all chief investigators and associate investigators, the clinical trial manager, research staff and chairs or proxy of each FNRG. This committee is the steering committee of the trial responsible for all aspects of the trial and making sure the trial is on course and meets every 2 months. A number of out session discussion also occur when needed.

### Composition of the data monitoring committee, its role and reporting structure {21a}

#### Data safety and monitoring board (DSMB)

An independent DSMB (none of whom are chief investigators or associate investigators) has been established to review the progress of the study and monitor adherence to the protocol, participant recruitment, outcomes, complications and other issues related to participant safety. They also monitor the assumptions underlying sample size calculations for the study and alert the investigators if they see substantial departures from the assumptions as the data accumulate.

The DSMB includes two clinician-researchers familiar with clinical trials, a statistician, and a First Nations clinical researcher. The DSMB met before recruitment commenced and 6 months following commencement of recruitment and will continue to meet at annual intervals and at its conclusion. Progress reports documenting serious adverse events will be provided to the DSMB 6-monthly. We have planned a formal interim analysis.

The DSMB will make recommendations as to whether the study should continue or be terminated and consider participant safety or other circumstances as grounds for early termination, including either compelling internal or external evidence of treatment differences or feasibility of addressing the study hypotheses (e.g. poor participant enrolment, poor adherence).

#### First Nation (Aboriginal and Torres Strait Islander) Reference Groups (FNRG)

Two First Nation Reference Groups (FNRG) have been established to provide oversight regarding culturally appropriate conduct of the research in their respective HREC-approved jurisdictions. Each FNRG consists of 7–10 members, of whom all are First Nations. One member each is an independent First Nations academic, with two [2] members from each of the dialysis units: FNRG [1] Top End – Royal Darwin Hospital, Nightcliff, Palmerston, Katherine and Tiwi Islands. FRRG [2] Central Australia - Alice Springs Hospital, Flynn Drive, Gap Road and Tenant Creek.

Of the two members from each site, ideally one will be male, and one will be female. The members can self-nominate or be nominated by or from First Nations communities and community-controlled services which are partners in this study. The FNRG will meet regularly throughout the period of the research conduct, and a representative will join the Trial Management Committee. If requested by the FNRG, the INFERR Clinical Trial Manager or the principal or chief investigator will attend the meetings to provide study progress reports.

### Adverse events reporting and harms {22}

#### Serious adverse events (SAEs)

A SAE is defined as any experience that:

- Results in death
- Is life threatening.
  - The term “life threatening” refers to an event in which the participant was at risk of death at the time of the event. It does not refer to an event which hypothetically may have caused death if it were more serious.
- Results in unexpected prolongation of existing hospitalisation
- Results in persistent or significant disability/incapacity
- Is a medically important event or reaction.

All SAEs that are considered related to the study drugs require reporting on the SAE form. If the SAE is attributable to disease progression, then this does not require expedited reporting in this trial, even when death is the outcome. The site PI is required to report any SAEs that occur at their site to the approving ethics committee in accordance with local guidelines; in addition, the site PI must adhere to any local institutional reporting requirements and study sponsor. Safety reporting will be in line with the National Health and Medical Research Council’s (NHMRC) Guidance: Safety monitoring and reporting in clinical trials involving therapeutic goods (2016) [53].

#### Suspected unexpected serious adverse reactions (SUSAR)

An adverse event that is both serious and unexpected (not listed in the product information and not attributable to disease progression) and related to the study drug meets the definition of a SUSAR. All SUSARs must be entered on the SAE CRF and reported to the sponsor or delegate within 24 h from the time of the site study team becoming aware of it. If the SUSAR is fatal or life threatening, the Sponsor will report to the TGA within 7 calendar days of becoming aware and any follow-up reports required within a further 8 calendar days, and for all others 15 calendar days of becoming aware.

The site PI is required to report any SUSARs that occur at their site to the approving ethics committee in accordance with local guidelines; in addition, the site PI must adhere to any local institutional reporting requirements. Safety reporting will be in line with the NHMRC’s Guidance: Safety monitoring and reporting in clinical trials involving therapeutic goods (2016) [53].

#### Adverse drug reactions (ADRs)

Investigators will be asked to report all suspected adverse drug reactions (regardless of severity or seriousness) which are thought to be related to study drugs in both intervention and control arms within the first 24 h post study drug infusion. These data will be collected routinely on CRFs.

#### Causality

##### Assessment of causality for study drugs

The principal investigator or their delegate is responsible for assessing the relationship to study drugs using clinical judgement and the following considerations:

- *Unrelated*: Evidence exists that the adverse event has an aetiology other than the study drugs. For SAEs, an alternative causality must be provided (e.g. pre-existing condition, underlying disease, intercurrent illness or concomitant medication).
- *Related*: There is reasonable possibility that the event may have been caused by the study drugs. It should be emphasised that ineffective treatment should not be considered as causally related in the context of adverse event reporting.

The degree of certainty with which an adverse event is attributable to treatment, or an alternative cause will be determined by how well the event can be understood in terms of:

- Temporal relationship with the administration of the treatment or cessation of treatment
- Reactions of a similar nature previously observed in the individual or others following treatment.

The relationship of the adverse event to treatment will be specified as follows:

- *Not related*In the PI’s opinion, there is not a causal relationship.
- *Unlikely*The temporal association between treatment and the adverse event is such that treatment is not likely to have any reasonable association.
- *Possibly*The adverse event could have been caused by treatment.
- *Probably*The adverse event follows a temporal sequence from the time of treatment and cannot be reasonably explained by the known characteristics of the participant’s clinical presentation/history.
- *Definitely*The adverse event follows a reasonable temporal sequence from the time of treatment or reappears when the treatment is repeated.

##### Non-expedited reporting of adverse events and adverse drug reactions

In addition to the expedited reporting described above, a summary of all adverse drug reactions (to any of the study drugs including SAEs) will be reported to the DSMB and HREC as per local guidelines.

##### Summary of expedited reporting of adverse events and adverse drug reactions

In summary, SAEs *not* thought to be related to the study drug do not need to be reported in this trial; however, this data will be collected for outcome measures. SAEs thought to be possibly, probably or definitely related to study drug (e.g. an anaphylactic reaction to IV iron) must be reported by the PI or CI, research nurse or their delegate to the sponsor (in this case the Clinical Trial Manager and CIs at Menzies) within 24 h of becoming aware of the event. Reporting to the HREC will be in accordance with local guidelines. If it is an expected side effect (i.e. one listed in the product information, such as allergic reaction or diarrhoea), it does not need to be reported to the TGA. If it is both *unexpected* and serious, it needs to be reported to the TGA within 7 days (fatal or life threatening) or 15 days (other).

### Frequency and plans for auditing trial conduct {23}

Study monitoring will be provided by the responsible monitor(s) at the Menzies School of Health Research (or designee) in accordance with the Monitoring Plan and “International Conference on Harmonisation of Technical Requirements for Registration of Pharmaceuticals for Human Use” Good Clinical Practice.

The responsible monitor will visit each study site at least once per year and will be allowed, on request, to inspect the various records (source documents, paper CRFs, eCRFs and other pertinent data) provided that participant confidentiality is maintained in accord with local requirements.

It will be the monitor’s responsibility to inspect the eCRFs throughout the study, to verify the adherence to the protocol and the completeness, consistency and accuracy of the data being entered on them. The monitor must verify that the participant received the study drug as randomised. The monitor should have access to laboratory test reports and other participant records needed to verify the entries on the eCRF. The investigators and site staff and teams agree to cooperate with the monitor to ensure that any problems detected in the course of these monitoring visits are resolved in a timely manner.

#### Safety

All the trial medications are licenced for use in Australia with established safety profiles and routinely used in clinical practice. A safety measure was written into the protocol to avoid putting participants at risk of either excessive or insufficient IV iron administration according to the current standards for haemodialysis patients. Prior to receiving any study treatment, participants’ blood results are reviewed for ferritin and TSAT levels; participants’ treatment varies based on the results of these blood levels:

- Assigned study drug will be administered for ferritin levels between ≥ 700 μg/L and ≤ 2000 μg/L and TSAT < 40% and ferritin < 700 μg/L and TSAT < 40% (Arm A) and no treatment for ferritin levels between ≥ 700 μg/L and ≤ 2000 μg/L and TSAT < 40% (Arm B) and rescue therapy for ferritin levels < 700 μg/L and TSAT< 40% (both arms)
- For both Arms A and B, if ferritin levels are < 700 μg/L *and* TSAT < 40%, “rescue treatment” will be administered for that month.
- If ferritin > 2000 μg/L and/or TSAT ≥ 40% for both Arms A and B, study treatment will be withheld and receive no treatment for that month.

### Plans for communicating important protocol amendments to relevant parties (e.g. trial participants, ethical committees) {25}

The investigator will inform the HRECs of the following:

- All protocol amendments, informed consent changes or revisions of other documents originally submitted for review.
- Serious and/or unexpected adverse events attributable to study drugs
- New information that may affect the safety of the participants or the proper conduct of the trial.
- Annual updates of study progress
- Termination of the study including provision of a final study report.

### Dissemination plans {31a}

#### Communicating trial results

The trial results will be communicated to all investigators and to IRG members prior to publication or presentation. The trial results will be presented at national and international scientific conferences. The trial results will also be submitted for publication to a peer-reviewed scientific journal, irrespective of the results. The FNRG will advise on participant and community feedback content and method of delivery. A plain-language summary of the trial results will be made available to individual participants.

## Discussion

The INFERR clinical trial will address significant uncertainty on the safety and efficacy of iron therapy in First Nations Australians with ESKD with hyperferritinaemia and evidence of iron deficiency. This will hopefully lead to the development of evidence-based guidelines. It will also provide the opportunity to explore the causes of hyperferritinaemia. Anaemia, and particularly, iron deficiency anaemia, is common among First Nations ESKD patients from the NT. The coexistence of hyperferritinaemia, inflammation and chronic infections complicates the diagnosis and management of iron deficiency in First Nations patients with CKD/ESKD which the trial will address [6].

The study will also evaluate the significant issues on the performance differences in ferritin measurement platforms to determine clinical decision limits in people with CKD/ESKD [6]. Identifying and evaluating patients with hepatic diseases such as hepatic steatosis and non-alcoholic fatty liver disease (NAFLD) as seen in high prevalent diabetes mellitus, metabolic syndrome states, dialysis dependence and dysmetabolic iron overload will help to adapt iron dosage and to monitor the safety of IV iron therapy in this setting [54–56].

Indiscriminate IV iron therapy has recently been shown to either trigger or aggravate NAFLD in dialysis patients which warrants the evaluation in the INFERR clinical trial [38, 54].

The use of FerriScans, recently shown to accurately measure hepatic iron load in dialysis patients will provide useful information for decision, choice and follow-up of iron therapy in patients with hyperferritinaemia and evidence or iron deficiency [34, 36].

## Trial status

Protocol version number: INFERR trial protocol 2.0

Began recruitment: 16 December 2020.

Approximate date when recruitment will be completed: 30 June 2023.

## Supplementary Information

**Additional file 1.**

**Additional file 2.**

**Additional file 3.**

**Additional file 4.**

**Additional file 5.**

## Abbreviations

ACTRN: Australian clinical trials registration number
ANZCTR: Australian New Zealand Clinical Trials Registry
AE: Adverse event
ADR: Adverse drug reaction
CA: Central Australia
CAHREC: Central Australia Human Research Committee
CKD: Chronic kidney disease
CARI: Australian and New Zealanders with Kidney Impairment
CRP: C-reactive protein
CI: Chief investigator
CONSORT: Consolidated Standards of Reporting Trials
CV: Cardiovascular
CWS: Clinical workstation
CRF: Case Report Form
CTN: Clinical Trial Notification
DRIVE: Dialysis Patients’ Response to IV Iron with Elevated Ferritin
DSMB: Data Safety and Monitoring Board
ESKD: End-stage kidney disease
ESA: Erythropoietin-stimulating agents
EPO: Erythropoietin
eCRF: Electronic Case Report Form
EDC: Electronic Data Capture
ERI: Erythropoietin Resistance Index
HbA1c: Glycated haemoglobin
Hb: Haemoglobin
HREC: Human Research Ethics Committees
HF: Heart failure
HRN: Hospital registration number
ICJME: International Committee of Medical Journal Editors
IL-1: Interleukin-1
IL-6: Interleukin-6
ID: Identification
IV: Intravenous
IR: Inter-quartile range
FNRG: First Nations Reference Group
KDIGO: Kidney Disease: Improving Global Outcomes
KDOQI: Kidney Disease Outcomes Quality Initiative
LFTs: Liver function tests
MHD: Maintenance haemodialysis
MCV: Mean corpuscular volume
MRI: Magnetic resonance imaging
MI: Myocardial infarction
MCHC: Mean corpuscular haemoglobin concentration
NHMRC: National Health and Medical Research Council
NICE: National Institute for Health and Care Excellence
NT: Northern Territory
PCIS: Primary Care Information System
PHRC: Percentage of hypochromic red blood cells
PIVOTAL: Proactive IV irOn Therapy in haemodiALysis patients
SOPs: Standard operating procedures
SD: Standard deviation
SAE: Serious adverse events
SUSAR: Suspected unexpected serious adverse reactions
REDCap: Research electronic data capture
RBC: Red blood cells
TGA: Therapeutic Goods Administration
TSAT: Transferrin saturation
TIBC: Total iron-binding capacity
TNF-α: Tumour necrosis alpha
