# Supplementary material for: INFERR-Iron infusion in haemodialysis study: INtravenous iron polymaltose for First Nations Australian patients with high FERRitin levels on haemodialysis—a protocol for a prospective open-label blinded endpoint randomised controlled trial
Source: Trials. 2021 Dec 2;22:868. doi: 10.1186/s13063-021-05854-w (PMC8641231; doi:10.1186/s13063-021-05854-w)
Supplement: Supplementary file 1 — Additional file 1. [file 13063_2021_5854_MOESM1_ESM.docx]

|  | PATIENT LABEL  Principal name  Other name(s)  D.O.B.  HRN  Sex  Address must be included if patient details are handwritten |
| --- | --- |
| **HAEMODIALYSIS IV IRON REGIMEN** |  |
|  | **Practice Points**   - Iron studies and CRP to attended monthly. - CRP 50mg/L and above DO NOT administer iron. - Administer as per the ‘Intravenous Iron on Haemodialysis NT Guideline’. - Record test dose reactions as per Intravenous Iron on Haemodialysis NT Guideline.   **Contact Renal Registrar or Consultant:**   - To prescribe initial regimen - When CRP 50mg/L and above - When iron study results not within range |

**HAEMODIALYSIS IV IRON REGIMEN**

| **Initial Test Dose Date** ___/___/___ | | | | | | |
| --- | --- | --- | --- | --- | --- | --- |
| **Regimen** | **Iron** | | | **IV Iron Dose** | | |
| **A** | TSAT equal to, or less than 40%  AND  Ferritin 700microg/L and less | | | **Iron 400mg monthly**  Administer 200mg in two consecutive doses or next treatment if haemodialysis missed following monthly iron results. | | |
| **B** | TSAT greater than 40%  AND/ OR  Ferritin above 700microg/L | | | **No iron administered for month**  Patient reverts to regimen A once TSAT and Ferritin within range.  **Document ‘no iron administered’ in administration record**  **After THREE consecutive results in this range notify renal registrar or consultant** | | |
| **Circle prescribed iron: Iron Polymaltose / Ferric Carboxymaltose / Other:** | | | | | | |
| **Prescriber:** Print Name Signature: Date: ___/___/___    (Valid for ONE year from date of prescription) | | | | | | |
| **Iron result monthly review** | | | | | | |
| **Date of Iron Results** | **% TSAT** | **Ferritin**  **(microg/L)** | **CRP**  **(mg/L)** | **Regimen** | **Registered Nurse Signatures** | |
|  |  |  |  |  |  |  |
|  |  |  |  |  |  |  |
|  |  |  |  |  |  |  |
|  |  |  |  |  |  |  |
|  |  |  |  |  |  |  |
|  |  |  |  |  |  |  |
|  |  |  |  |  |  |  |
|  |  |  |  |  |  |  |
|  |  |  |  |  |  |  |
|  |  |  |  |  |  |  |
|  |  |  |  |  |  |  |
|  |  |  |  |  |  |  |

| **TEST DOSE ONLY** | | **Date:** ___/___/___ **Nurse Signatures:** ___________ ___________ **Reaction: Yes 🞎 No 🞎** | | | | | | | |
| --- | --- | --- | --- | --- | --- | --- | --- | --- | --- |
|  |  | |  |  | |  |  |  | |
| **Month** | **Date** | | **First Dose** | **Signatures** | | **Date** | **Second Dose** | **Signatures** | |
|  |  | |  |  |  |  |  |  |  |
|  |  | |  |  |  |  |  |  |  |
|  |  | |  |  |  |  |  |  |  |
|  |  | |  |  |  |  |  |  |  |
|  |  | |  |  |  |  |  |  |  |
|  |  | |  |  |  |  |  |  |  |
|  |  | |  |  |  |  |  |  |  |
|  |  | |  |  |  |  |  |  |  |
|  |  | |  |  |  |  |  |  |  |
|  |  | |  |  |  |  |  |  |  |
|  |  | |  |  |  |  |  |  |  |
|  |  | |  |  |  |  |  |  |  |

**Administration record**

Page 1 of 1

HR 0000

MR000.0065

Page 1 of 2
