# Supplementary material for: INFERR-Iron infusion in haemodialysis study: INtravenous iron polymaltose for First Nations Australian patients with high FERRitin levels on haemodialysis—a protocol for a prospective open-label blinded endpoint randomised controlled trial
Source: Trials. 2021 Dec 2;22:868. doi: 10.1186/s13063-021-05854-w (PMC8641231; doi:10.1186/s13063-021-05854-w)
Supplement: Supplementary file 2 — Additional file 2. [file 13063_2021_5854_MOESM2_ESM.docx]

MR???.??

|  | 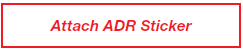  Prescriber to print patient name and check label correct  _______________________________________________  **IRON POLYMALTOSE ONLY** | 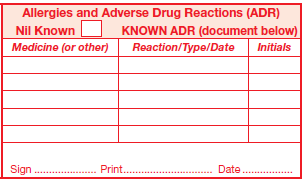 | Patient Label  Principal name  Other name (s)  D.O.B.  HRN  Sex  Address must be documented if details hand written |
| --- | --- | --- | --- |
| **HAEMODIALYSIS INFERR IV IRON TRIAL REGIMEN** |  |  |  |

|  |
| --- |

| **Practice Points**   - Iron studies and CRP attended monthly. | | | | | | | Contact Renal Registrar or Consultant: - To prescribe initial regimen   - When CRP 50mg/L and above - When iron study results not within range | | | | | | | | | | | | - Administer iron polymaltose as per the ‘Intravenous Iron on Haemodialysis NT Guideline’. | | | | |
| --- | --- | --- | --- | --- | --- | --- | --- | --- | --- | --- | --- | --- | --- | --- | --- | --- | --- | --- | --- | --- | --- | --- | --- |
| - CRP 50mg/L and above DO NOT administer iron. | | | | | | |  |  |  |  |  |  |  |  |  |  |  |  | - Record test dose reactions as per the guideline. | | | | |
|  | | | | | | | | | | | | | | | | | | | | | | | |
| **Arm A** | | | | | | | | | | |  | **Arm B** | | | | | | | | | | | |
| **1A** | TSAT equal to, or less than 40%  AND  Ferritin 2000microg/L and less | | | **Iron 400mg monthly**  Administer 200mg in two consecutive haemodialysis sessions following monthly iron results. | | | | | | |  | **1B** | TSAT equal to, or less than 40%  AND  Ferritin 700 – 2000microg/L | | | | **No iron administered**  **Record ‘no iron administered’ in administration record** | | | | | | |
| **2A** | TSAT greater than 40%  AND/ OR  Ferritin above2000 microg/L | | | **No iron administered for month**  Patient reverts to **1A** once TSAT and Ferritin within range.  **Record ‘no iron administered’ in administration record**  **After THREE consecutive results in this range notify Renal Registrar or Consultant** | | | | | | |  | **2B** | TSAT equal to, or less than 40%  AND  Ferritin less than 700microg/L | | | | **Iron 400mg monthly**  Administer 200mg in two consecutive haemodialysis sessions following monthly iron results.  Patient reverts to **1B** when TSAT and Ferritin within range. | | | | | | |
|  |  |  |  |  |  |  |  |  |  |  |  | **3B** | TSAT greater than 40%  AND/ OR  Ferritin above 2000microg/L | | | | **No iron administered for month**  Patient reverts to **1B** once TSAT and Ferritin within range.  **Record ‘no iron administered’ in administration record**  **After THREE consecutive results in this range notify Renal Registrar or Consultant** | | | | | | |
| **Prescriber**: Print Name ________________ Signature: __________________ Date: ___/___/___ (Valid for ONE year from date of prescription) Pager / Contact number: __________  Randomisation Arm □ A □ B Randomisation Date: ___/___/___ Study ID: ____________ | | | | | | | | | | | | | | | | | | | | | | | |
| Initial Test Dose Administered on ___/___/___ TEST DOSE ONLY Date: ___/___/___ Nurse Signatures: _________________ ________________ Reaction: Yes □ No □ | | | | | | | | | | | | | | | | | | | | | | | |
| Nurse 1 Nurse 2 | | | | | | | | | | | | | | | | | | | | | | | |
| **Iron Results** | | **% TSAT** | **Ferritin (microg/L)** | | **Hb (g/L)** | **CRP (mg/L)** | | **Arm** | **Nurse Sign** | | | | | **Month** | **First Dose** | **Nurse Sign** | | | | **Date** | **Second Dose** | **Nurse Sign** | |
| ___/___/___ | |  |  | |  |  | |  | Nurse 1 | Nurse 2 | | | |  |  | Nurse 1 | | Nurse 2 | | ___/___/___ |  | Nurse 1 | Nurse 2 |
| ___/___/___ | |  |  | |  |  | |  | Nurse 1 | Nurse 2 | | | |  |  | Nurse 1 | | Nurse 2 | | ___/___/___ |  | Nurse 1 | Nurse 2 |
| ___/___/___ | |  |  | |  |  | |  | Nurse 1 | Nurse 2 | | | |  |  | Nurse 1 | | Nurse 2 | | ___/___/___ |  | Nurse 1 | Nurse 2 |
| ___/___/___ | |  |  | |  |  | |  | Nurse 1 | Nurse 2 | | | |  |  | Nurse 1 | | Nurse 2 | | ___/___/___ |  | Nurse 1 | Nurse 2 |
| ___/___/___ | |  |  | |  |  | |  | Nurse 1 | Nurse 2 | | | |  |  | Nurse 1 | | Nurse 2 | | ___/___/___ |  | Nurse 1 | Nurse 2 |
| ___/___/___ | |  |  | |  |  | |  | Nurse 1 | Nurse 2 | | | |  |  | Nurse 1 | | Nurse 2 | | ___/___/___ |  | Nurse 1 | Nurse 2 |
| ___/___/___ | |  |  | |  |  | |  | Nurse 1 | Nurse 2 | | | |  |  | Nurse 1 | | Nurse 2 | | ___/___/___ |  | Nurse 1 | Nurse 2 |
| ___/___/___ | |  |  | |  |  | |  | Nurse 1 | Nurse 2 | | | |  |  | Nurse 1 | | Nurse 2 | | ___/___/___ |  | Nurse 1 | Nurse 2 |
| ___/___/___ | |  |  | |  |  | |  | Nurse 1 | Nurse 2 | | | |  |  | Nurse 1 | | Nurse 2 | | ___/___/___ |  | Nurse 1 | Nurse 2 |
| ___/___/___ | |  |  | |  |  | |  | Nurse 1 | Nurse 2 | | | |  |  | Nurse 1 | | Nurse 2 | | ___/___/___ |  | Nurse 1 | Nurse 2 |
| ___/___/___ | |  |  | |  |  | |  | Nurse 1 | Nurse 2 | | | |  |  | Nurse 1 | | Nurse 2 | | ___/___/___ |  | Nurse 1 | Nurse 2 |
| ___/___/___ | |  |  | |  |  | |  | Nurse 1 | Nurse 2 | | | |  |  | Nurse 1 | | Nurse 2 | | ___/___/___ |  | Nurse 1 | Nurse 2 |
| **HAEMODIALYSIS INFERR IV IRON TRIAL REGIMEN** | | | | | | | | | | | | | | | | | | | | | | | |
