# Supplementary material for: INFERR-Iron infusion in haemodialysis study: INtravenous iron polymaltose for First Nations Australian patients with high FERRitin levels on haemodialysis—a protocol for a prospective open-label blinded endpoint randomised controlled trial
Source: Trials. 2021 Dec 2;22:868. doi: 10.1186/s13063-021-05854-w (PMC8641231; doi:10.1186/s13063-021-05854-w)
Supplement: Supplementary file 3 — Additional file 3. [file 13063_2021_5854_MOESM3_ESM.docx]

**INFERR Study Specific Procedure**

**Laboratory Sampling**

**Version 1.0 dated 30^th^ June 2021**

| Name | INFERR Trial SSP for collection, handling, and processing of laboratory samples |
| --- | --- |
| Revision Number | 1.0 |
| Owner | A/Prof Sandawana William Majoni |
| Implementation date | 01/08/2021 |
| Last review date | 31/07/2021 |
| Approver |  |

**Contents**

[**1** **PURPOSE** 2](#_Toc78642169)

[**2** **RESPONSIBILITY, SCOPE AND APPLICABILITY** 2](#_Toc78642170)

[**3** **INFERR RECRUITMENT LOCATIONS** 2](#_Toc78642171)

[**4** **PROCEDURES** 2](#_Toc78642172)

[**4.1** **Brief overview** 2](#_Toc78642173)

[**4.2** **Ordering, collecting and initial transport of bloods:** 3](#_Toc78642174)

[4.2.1 Pathology specimen form 3](#_Toc78642175)

[4.2.2 Process for ordering and collection of specimens 4](#_Toc78642176)

[4.2.3 Labelling of aliquots 4](#_Toc78642177)

[**4.3** **Comparison of ferritin concentration measurement methods** 4](#_Toc78642178)

[**4.4** **Table 1: INFERR - Overview of Sample Collection** 5](#_Toc78642179)

[**4.5** **Table 2: Territory Pathology - Specimen processing, storage and shipment** 6](#_Toc78642180)

[**4.6** **Transporting frozen serum to RDH/ASH** 7](#_Toc78642181)

[4.6.1 Katherine District Hospital 7](#_Toc78642182)

[4.6.2 Tennant Creek Hospital 7](#_Toc78642183)

[**4.7** **Transporting frozen serum specimen’s interstate – 3 monthly** 7](#_Toc78642184)

[**4.8** **Hepcidin – Menzies** 8](#_Toc78642185)

[**4.9** **Certification to handle and transport biological substances and dry ice** 9](#_Toc78642186)

[**4.10** **Disposal of samples** 9](#_Toc78642187)

[**5** **FAQ** 9](#_Toc78642188)

[**6** **GLOSSARY OF TERMS** 10](#_Toc78642189)

[**7** **ABBREVIATIONS** 11](#_Toc78642190)

# **PURPOSE**

The purpose of this Standard Operating Procedure (SOP) is to document the procedure for the collection, handling, and transport of biological samples for the INFERR clinical trial.

# **RESPONSIBILITY, SCOPE AND APPLICABILITY**

This SOP applies to Northern Territory (NT) Renal service staff, Menzies School of Health Research (MSHR) staff, investigators and NT Territory Pathology staff who undertake any of the following roles in INFERR clinical trial:

- NT Renal units’ staff are responsible for collecting specimens at the scheduled times every month and three monthly depending on the type of tests as part of routine standard clinical care
- INFERR Investigators at Menzies, Darwin, and Alice Springs plan with the NT Territory Pathology and Menzies laboratory staff, the handling and processing of samples
- Members of the INFERR research team in Darwin and Alice Springs who have been delegated responsibility for the collection, handling and/or transport of biological samples.
- Menzies laboratory staff handling and processing the samples sent to the Menzies laboratory from NT Territory laboratory
- Royal Darwin Hospital (RDH) & Alice Springs Hospitals’ (ASH)Territory Pathology (including Katherine and Tennant Creek) staff who are processing, storing, and shipping samples for the INFERR clinical trial

# **INFERR RECRUITMENT LOCATIONS**

The INFERR trial will recruit 576 participants over 3.5 years from participating dialysis centres.

| **Top End:**   - Royal Darwin Hospital, - Nightcliff, - Palmerston, - Katherine, - Tiwi Islands | **Central Australia:**   - Alice Springs Hospital, - Gap Road, - Flynn Drive - Tennant Creek. |
| --- | --- |

# **PROCEDURES**

## **Brief overview**

Monthly or three-monthly routine standard clinical care blood specimens will be collected by the renal nurses in the participating dialysis units (Table 1). INFERR specific request forms and labels are used to identify the collection tubes used for each participant for the collection of these samples (section 4.3). The specimens will be processed by Territory Pathology in Darwin, Alice Springs Hospitals. Katherine and Tennant Creek as per the NT Territory Laboratories procedures. When samples are couriered from participating dialysis units to either RDH or ASH, a copy of the courier form should be retained by the site staff and submitted to a member of the INFERR team either via email or in person at the next available opportunity

The additional samples outlined in Table 1 are mainly to address the relationship between high ferritin and other measures of iron levels, inflammation, and a marker of the metabolic syndrome. These will be collected within 6 months of the participate being recruited into the trial and at 12 months post recruitment. A number of these samples will be routinely processed in the NT Territory Pathology Laboratory as they are part of routine requests as clinically indicated (markers of iron studies except hepcidin). However, some samples will be processed at the Menzies laboratory (Hepcidin), South Australian Laboratory (the inflammatory markers) and Melbourne Laboratory (Adiponectin). The laboratory linked to each dialysis unit will be asked to freeze and store some samples as per Table 2.

Samples must be handled in accordance with the requirements specified in the study protocol.

- Select the appropriate container to collect and store the sample (Table 1)
- Accurately label the sample using INFERR specific sticky labels
- Ensure that the immediate sample handling (methods and time requirements) complies with the study protocol and Territory Pathology Specimen Collection Guide

Stipulates the required tubes and handling

## **Ordering, collecting and initial transport of bloods:**

### **Pathology specimen form**

Require 8x copies of form to be placed with aliquots

**Instructions included with the path form:**

**FBC** (**RHB** [Percentage hypochromic RBC and Reticulocyte haemoglobin] is done with FBC)

**CYTOK (**Interleukin 1, Interleukin 6 and Tumour Necrosis Factor alpha)

- **Within 24 hours of collection** Freeze two serum aliquots in the Sendaway freezer INFERR bin, with 2 forms

**ADIPO** (Adiponectin)

- **Within 24 hours of collection** Freeze two serum aliquots in the Sendaway freezer INFERR bin, with 2 forms

**STFR** (Soluble transferrin receptor)

- - Send one serum aliquot to RBH cold as a routine Sendaway

**HPCD** (Hepcidin)

- RDH Only - Put one SST in the Reception fridge Menzies bin, with form. The hepcidin can also be aliquoted and stored at RDH and ASH. The samples could physically be pick-up from RDH monthly or 3 monthly and the specimens could be shipped from ASH directly to Menzies 3 monthly
- KDH, TCH & ASH: **Within 24 hours of collection** Freeze one serum aliquot in the send away freezer INFERR bin, with 1 form

### **Process for ordering and collection of specimens**

- The pathology request form developed by Territory Pathology in consultation with the INFERR team is placed in the participants renal unit file approx. 1 week prior to the routine monthly blood collection.
- The Path form stipulates the required tubes (Table 1), labelling (INFERR specific labels) and handling requirements (4.3).
- The renal nurse collects the blood as described in the path request form, record time & date of collection on the path form and keeps samples cool until transport to the Laboratory.
- The bloods are couriered to TP (RDH, ASH, KDH, TCH) for receipt, processing, disposal or storage and transferring (Table 2) as per routine practice.
- On arrival at the laboratory the path request form is dated and time stamped. The path form is copied x8. One copy will be placed with the hepcidin for Menzies.

### **Labelling of aliquots**

Tumour Necrosis Factor (TNF) alpha, Interleukin-1(IL-1) Interleukin-6 (IL-6), Adiponectin and hepcidin will be labelled at sites as per routine practice.

Menzies will use a brady label to store the hepcidin aliquot, the label will contain the following details:

INFERR

ID: _ _ - _ _ _ _

Date of collection: DDMMMYYYY

SERUM

Aliquots will be stored in the minus 80 freezer located in the SPA, shelf 4, rack 3. Rack position is top right. Jennie from Lab Support will transfer samples or box to the main laboratory on request.

Day and time of freezing is recorded on the path form and the information is recorded in the specimen accountability log.

## **Comparison of ferritin concentration measurement methods**

Ferritin is collected routinely each month as part of patient’s clinical care, this measurement forms part of the participants iron studies. To determine the comparability of ferritin levels across the Abbott 7 K59 ARCHITECT versus the Vitros Ortho XT7600 approximately 150 participants on the INFERR trial will have the some blood specimen processed using the two different assays. This will allow formal assessment of comparability of ferritin levels across these two relevant testing methods currently in use across the Northern Territory. This validation of the two testing methods is crucial for the safety and well-being of trial participants. No additional blood is collected from the participant.

## **Table 1: INFERR - Overview of Sample Collection**

| **Type** | **STANDARD CLINICAL CARE*** | **STANDARD CLINICAL CARE*** | **OTHER ROUTINE ADDITIONAL*** |
| --- | --- | --- | --- |
| **Frequency** | Monthly | 3 Monthly | Baseline (window 1 to 6 months) and  12 months (window +/- 2 weeks) |
| **Blood test** | Hb  CRP  FBC  LFT  Iron studies (including iron, ferritin, transferrin, TSAT) | PTH  HbA1C | *Thyroid Function Tests (TFT)  *Vitamin B12  *Folate (baseline only)  ****Inflammatory markers**  Tumour Necrosis Factor (TNF) alpha  Interleukin-1(IL-1) Interleukin-6 (IL-6)  ****Other markers of iron levels**  Percentage hypochromic red cells (PHRC)  Reticulocyte haemoglobin content (CHr)  Soluble transferrin factor (sTfR)  Hepcidin  ****Markers of the metabolic syndrome**  Adiponectin |
| **Collection**  **Tube** | Heparin (light green Vacutainer)  X1 3.0ml EDTA (lavender Vacutainer) | X1 3.0ml EDTA (lavender Vacutainer) | *x1 Heparin (light green Vacutainer)  **x1 5.0ml Gold SST Gel |

NOTE: Blood specimens must be taken before dialysis

## **Table 2: Territory Pathology - Specimen processing, storage and shipment**

| **Frequency** | **Blood test** | **Time to storage** | **Processing & forms** | **Storage requirements** | **Shipment – Referred frequency** |
| --- | --- | --- | --- | --- | --- |
| Monthly | Hb, CRP, FBC, LFT and Iron studies (including iron, ferritin, transferrin, TSAT) | N/A | Routinely processed  Results (Labtrak) | Minimum storage as per usual lab practice then destroyed | N/A |
| 3 Monthly | PTH & HbA1C | N/A | Routinely processed  Results (Labtrak) | Minimum storage as per usual lab practice then destroyed | N/A |
| Baseline & 12 months | Thyroid Function Tests (TFT)  Vitamin B12  Folate (baseline only) | N/A | Routinely processed  Results (Labtrak) | Minimum storage as per usual lab practice then destroyed | N/A |
| Baseline & 12 months | Percentage hypochromic red cells (PHRC)  Reticulocyte haemoglobin content (CHr)  Soluble transferrin factor (sTfR) | N/A | Routinely processed  Results (Labtrak) | Minimum storage as per usual lab practice then destroyed | N/A |
| Baseline & 12 months | Tumour Necrosis Factor (TNF) alpha *  Interleukin-1(IL-1) Interleukin-6 (IL-6)* | Within 24 hours of collection | Freeze two serum aliquots in the Sendaway freezer INFERR bin, with 2 forms | Minus 70℃ to 80℃ | Referred 3 monthly  Transfer frozen to SA Pathology at WHC Campus contact Dr Tony Ferrante T 08 8161 6637 M 0423 025 582 Laboratory for processing then destroyed |
| Baseline & 12 months | Adiponectin* | Within 24 hours of collection | Freeze two serum aliquots in the Sendaway freezer INFERR bin, with 2 forms | Minus 70℃ to 80℃ | Referred 3 monthly  Transfer frozen to Vic, St Vincent’s Laboratory Prof Macisalac for processing then destroyed |
| Baseline & 12 months | Hepcidin** | Within 24 hours of collection | Sites: Freeze one serum aliquot in the Sendaway freezer INFERR bin  RDH only-Put one SST in the Reception fridge Menzies bin, with form. | Menzies – Aliquot, label and store in Minus 70℃ to 80℃.  Processed in batches and then destroyed. | Referred 3 monthly to Menzies, Darwin. |

*Transfer in batches from site to RDH for storage and eventual shipment

## **Transporting frozen serum to RDH/ASH**

Standard and routine and additional clinical specimens: Where there is an availability of dry ice this will be preference for the transfer of frozen serum. Local labs will be responsible for organising and shipping aliquots centrally to RDH or ASH.

Additional clinical specimens (CYTOK, ADIPO, STFR): RDH and ASH will be responsible for shipment of frozen serum to SA pathology and St Vincent Pathology (Vic).

Additional clinical specimens (HPCD): Menzies will cover the costs of shipment for the frozen hepcidin aliquot, Menzies will liaise with ASH to assist with organisation of the shipment to the Menzies lab.

### Katherine Hospital

The frozen serum for CYTOK, ADIPO, STFR and HPCD will require transfer to RDH, this will be done every 3 months as per standard laboratory processes/procedures.

### Tennant Creek Hospital

The frozen serum for CYTOK, ADIPO, STFR and HPCD will require transfer to ASH, this will be done every 3 months as per standard laboratory processes/procedures.

## **Transporting frozen serum specimen’s interstate – 3 monthly**

- Transport standards: Please refer to section 5.3
- Certifications: Please refer to section 5.4.

### **Delivery address**

**Cytokines (IL-1, IL-6, TNF)** every 3 months will be transferred to the following laboratory for processing:

Referred to Immunopathology

SA Pathology at WCH Campus

Contact Dr Tony Ferrante T 08 8161 6637 M 0423 025 582

**Adeponection** every 3 months will be transferred to the following laboratory for processing:

Referred to

St Vincent’s Laboratory

Vic

(Still under discussion)

**Hepcidin** every 3 months and will be picked up by INFERR researcher every month

Top End

Menzies School of Health Research

Jane Nelson 0416 265 448 or Libby Hoppo 0439380692

[inferr@menzies.edu.au](mailto:inferr@menzies.edu.au)

Central Australia

Menzies School of Health Research

Jessica Graham 0428074331

[Jessica.graham@menzies.edu.au](mailto:Jessica.graham@menzies.edu.au)

## **Hepcidin – Menzies**

The hepcidin specimen is centrifuge at local laboratory. For sites other than RDH the serum is transferred into one aliquot and transferred into the INFERR sendaway minus 70 to 80 freezer. Shipments from sites will occur 3 monthly to coincide with other frozen specimens.

For hepcidin samples, all samples that are to be stored refrigerated at RDH or ASH prior to picking up by Menzies INFERR research staff (refer to 4.6 for Menzies contact). A copy of the path form must be placed with the sample.

**Standards for transporting of samples**

Samples will be transported to the respective laboratories (see section 4.3) in batches at four specified time points in the study – 3 months after the recruitment of participant # 100, 200, 300, 400 and 576. Samples will be identified by their INFERR study number and local laboratory specimen ID number.

According to Australian legislation (Civil Aviation Act 1988), transporting of dangerous goods such as biological and infectious products, dry ice and liquid nitrogen by air must be packed by certified staff. Failure to do so could result in the air carrier refusing to accept the package or financial penalties or a jail term.

Territory Pathology has a number of trained support staff who can assist INFERR staff members with shipping. These staff members have completed the Safe Transport of Infectious Substances by Air (International Air Transport Association; IATA) course. Contact the appropriate Territory Pathology laboratory staff for assistance (see Contacts, Section 6).

Where transport of samples is delegated, it remains the Investigator’s responsibility to ensure all procedures and regulations are adhered to.

The investigator must:

- Ensure that ethical approval and participant consent is in place prior to transporting biological samples to Menzies School of Health Research laboratories or other laboratories.
- Ensure specimens are handled in accordance with local requirements as written in the INFERR protocol, Territory Pathology Specimen Collection Guide and as per standard clinical practice.
- Ensure specimens are packed and shipped in accordance with local requirements as written in the protocol and according to IATA and Civil Aviation Safety Authority (CASA) requirements, including:
- Ensure that all study staff, who handle or ship biological substances (i.e., who enclose the goods in packaging, or mark or label the consignment, or prepare a shipper's declaration) are certified hold a current certificate gained through completion of an IATA/CASA approved Certified Dangerous Goods Packaging Course (see section 4.5). Where research personnel do NOT hold current certification, arrangements for biological substance / dry ice shipment must be made with certified staff (as noted above, contact the INFERR Clinical Trial Manager).
- Ensure that a valid export permit is in place (where required). Contact the designated Territory Pathology laboratory for assistance, if required.
- Ensure that documentation (e.g., Receipts, shipping records, order forms, proformas etc.) related to handling and shipment of biological specimens is maintained and filed in the respective INFERR Investigator Site File.

## **Certification to handle and transport biological substances and dry ice**

• CASA Regulations have defined categories of personnel who should attend training and the subject matter in which they must be qualified. These regulations are mandatory and legally binding, and consequently must be adhered to in full.

• All INFERR study and Territory Pathology staff, who handle or ship biological substances (i.e., who enclose the goods in packaging, or mark or label the consignment, or prepare a shipper's declaration) hold a current certificate gained through completion of a Civil Aviation Safety Authority (CASA)- approved Certified Dangerous Goods Packaging Course – see

- https://www.casa.gov.au/standard-page/dangerous-goods-courses
- https://www.casa.gov.au/standard-page/organisations-offering-shipperscourses-infectious-substances-diagnostic-specimens-and-dry-ice).

• The Civil Aviation Safety Authority (CASA) Certified Dangerous Goods Packaging Course can be done by any media and must be recorded on the respective training log as per good clinical practice requirements and that copies of certificates are kept in the respective Site Investigator File.

• Re-certification is required every two years. Certificates and any training records must be kept for a minimum period of 36 months from the most recent training completion date, and must be made available, upon request by regulatory authority and CASA.

## **Disposal of samples**

Human research waste must be disposed of according to the “Menzies Laboratory Waste Disposal Chart”, available on the Menzies intranet. For all samples processed at Territory Pathology Laboratories and other laboratories, they should be destroyed after the analysis is completed within the specified period for the laboratory. No samples are too be stored beyond this period.

# **FAQ**

How are specimens sent to the laboratory?

- All specimens including the samples for additional tests are transported to the Territory Pathology as per routine practice/procedure from the renal units.

How about additional tests?

- All additional samples are transported to the laboratory in the same way as routine samples using routine procedures.

How can specimens be identified by the staff as research specimens?

- In the laboratory, INFERR specimens are on INFERR specific request forms and labels on the specimen tubes for the additional samples.
- Samples for routine specimens are on the usual routine requests.

How are there any special handling requirements?

- Some additional samples will need to be frozen at -80 degrees Celsius, see below.
- Samples for hepcidin analysis will need to be transferred to the MSHR for processing
- Samples that are for transfer to South Australia and Melbourne are frozen and will be transported to these laboratories from Territory Laboratories in batches
- Additional samples that cannot be processed in ASH are transported to RDH
- All additional samples from Katherine and Tenant Creek will be transport to RDH and ASH respectively.

# **GLOSSARY OF TERMS**

| INFERR | INFERR-Iron Infusion in Haemodialysis Study: Intravenous Iron Polymaltose for Aboriginal and Torres Strait Islander Patients with High Ferritin Levels on Haemodialysis: A Prospective Open-Label Blinded Endpoint Randomised Controlled Trial |
| --- | --- |
| End Stage Kidney Disease | the final, permanent stage of chronic kidney disease, where kidney function has declined to the point that the kidneys can no longer function on their own. |
| Ferritin | a protein produced in mammalian metabolism which serves to store iron in the tissues. It is used as a surrogate maker of iron levels in the body together with TSAT |
| Transferrin Saturation | measured as a percentage is the value of serum iron divided by the total iron-binding capacity of the available transferrin, the main protein that binds iron in the blood, this value tells a clinician how much serum iron is bound. It is used as a surrogate marker of iron stores together with ferritin. |
| C Reactive Protein | is an annular (ring-shaped) pentameric protein found in blood plasma, whose circulating concentrations rise in response to inflammation. It is an acute-phase protein of hepatic origin that increases following interleukin-6 secretion by macrophages and T cells |
| Hepcidin | is a protein that in humans is encoded by the HAMP gene and is a key regulator of the entry of iron into the circulation in mammals. |
| Inflammatory markers | a disparate set of biomarkers that are used clinically to assess a patient for: presence/absence of an active inflammatory disease process |
| Adiponectin | the most abundant peptide predominantly secreted by adipocytes, whose reduction plays a central role in obesity-related diseases, including insulin resistance/type 2 diabetes and cardiovascular disease. |
| Tumour necrosis factor (TNF, cachexin, or cachectin; often called tumour necrosis factor alpha or TNF-α) | is a cytokine – a small protein used by the immune system for cell signalling. If macrophages (certain white blood cells) detect an infection, they release TNF to alert other immune system cells as part of an inflammatory response. |
| The Interleukin-1 family (IL-1 family) | is a group of 11 cytokines that plays a central role in the regulation of immune and inflammatory responses to infections or sterile insults. |
| The Interleukin-1 family (IL-1 family) | is a group of 11 cytokines that plays a central role in the regulation of immune and inflammatory responses to infections or sterile insults. |
| Interleukin 6 (IL-6) | is an interleukin that acts as both a pro-inflammatory cytokine and an anti-inflammatory myokine. |
| Percentage hypochromic red cells | defined as red blood cells with a haemoglobin concentration of less than 28 g/dl) has been shown to detect insufficient marrow iron supply with a fairly good accuracy |
| The reticulocyte haemoglobin content (CHr) | provides an indirect measure of the functional iron available for new red blood cell production over the previous 3-4 days. Measurement of reticulocyte haemoglobin content in peripheral blood samples is useful for diagnosis of iron deficiency in adults |
| Soluble transferrin factor | conventionally refers to the cleaved extracellular portion of transferrin receptor 1 that is released into serum. Blood testing of the soluble transferrin receptor (sTfR) is used as a measure of functional iron status and the investigation of iron deficiency anaemia. |
| Essential documents | Essential documents are “Documents which individually and collectively permit evaluation of the conduct of a study and the quality of the data produced”. |
| Case Report Form (CRF) | A paper or electronic data collection document used in human research. It is a tool used to collect data on each study participant. The CRF consists of CRF pages. |
| Good Clinical Practice (GCP) | A standard for the design, conduct, performance, monitoring, auditing, recording, analyses, and reporting of clinical trials that provides assurance that the data and reported results are credible and accurate, and that the rights, integrity, and confidentiality of trial subjects are protected. |
| International Conference on Harmonisation (ICH) | International Conference on Harmonisation of Technical Requirements for Registration of Pharmaceuticals for Human Use is a joint initiative involving both regulators and research-based industry focusing on the technical requirements for medicinal products containing new drugs. |
| Investigator | An individual responsible for the conduct of a study, ensuring that the study complies with GCP guidelines.  • If a study is conducted by a team of individuals at a study site, the investigator is the responsible leader of the team and may be called the Chief Investigator (CI). In this instance they may delegate tasks to other team members.  • If a study is conducted at more than one study site, the Investigator taking overall responsibility for the study and for the coordination across all sites is known as the Chief Investigator (CI); the Principal Investigator at each site will retain responsibility for the conduct of the study at their site |
| Menzies | This term is used to encompass all staff from Menzies School of Health Research |
| Standard Operating Procedure (SOP) | Detailed, written instructions to achieve uniformity of the performance of a specific function. |

# **ABBREVIATIONS**

| CRF | Case report form |
| --- | --- |
| ESKD | End stage kidney disease |
| TSAT | Transferrin saturation |
| Hb | Haemoglobin |
| FBC | Full blood count |
| LTF | Liver function tests |
| Chr | reticulocyte haemoglobin content |
| PHRC | Percentage hypochromic red cells |
| IL-6 | interleukin 6 |
| IL-1 | interleukin-1 |
| TNF | Tumour necrosis factor |
| sTfR | Soluble transfer factor |
| TFT | Thyroid function test |

**INFERR Clinical Trial Study Staff /Territory Pathology Contacts**

INFERR Top End staff

| Title | Name | Email | Telephone |
| --- | --- | --- | --- |
| INFERR Chief Investigator | A/Prof Sandawana William Majoni | [william.majoni@nt.gov.au](mailto:william.majoni@nt.gov.au)  [william.majoni@menzies.edu.au](mailto:william.majoni@menzies.edu.au) | 0419931089 |
| Operational research support manager | Jane nelson | [Jane.nelson@menzies.edu.au](mailto:Jane.nelson@menzies.edu.au) | 0416265448 |
| INFERR Clinical Trial Manager | TBA | TBA | TBA |
| INFERR Research Nurse (TE) | Elizabeth (Libby) Hoppo | [inferr@menzies.edu.au](mailto:inferr@menzies.edu.au) | +0439380692 |
| INFERR Research Assistant (TE) | Stephanie Long | [Stephanie.long@menzies.edu.au](mailto:Stephanie.long@menzies.edu.au) |  |

INFERR Central Australia Staff

| Title | Name | Email | Telephone |
| --- | --- | --- | --- |
| INFERR Chief Investigator | A/Prof Sajiv Cherian | [Cherian.sajiv@nt.gov.au](mailto:Cherian.sajiv@nt.gov.au) | 0410186676 |
| INFERR Clinical Trial Manager | TBA | TBA | TBA |
| INFERR Research Nurse (CA) | Jessica Graham | [Jessica.graham@menzies.edu.au](mailto:Jessica.graham@menzies.edu.au) | 0428074331 |
| INFERR Research Assistant (CA) | TBA | TBA | TBA |

Territory Pathology Staff - RDH

| Title | Name | Email | Telephone |
| --- | --- | --- | --- |
|  | Robert McFarlane | [Robert.McFarlane@nt.gov.au](mailto:Robert.McFarlane@nt.gov.au) |  |
| Director, Territory Pathology | Dr Geetha Rathnayake | [Geetha.Rathnayake@nt.gov.au](mailto:Geetha.Rathnayake@nt.gov.au) | 08 8922 8079 |
|  | Jenna Ashford | [Jenna.ASHFORD@nt.gov.au](mailto:Jenna.ASHFORD@nt.gov.au) |  |
| Specimen Reception in charge scientist | Leonne Sach | [Leonne.Sach@nt.gov.au](mailto:Leonne.Sach@nt.gov.au) |  |
| Tech staff (shipping) | TBA | TBA | TBA |
|  |  |  |  |

Territory Pathology Staff – ASH

| Title | Name | Email | Telephone |
| --- | --- | --- | --- |
| Supervising Scientist Chemical Pathology | Lyn Hocking | [lyn.hocking@nt.gov.au](mailto:lyn.hocking@nt.gov.au) | 08 8951 7753 |
| Tech staff (shipping) | TBA | TBA | TBA |
|  |  |  |  |

Territory Pathology Staff – KDH

| Title | Name | Email | Telephone |
| --- | --- | --- | --- |
| Lab Manager | Tinashe Mutize | [Tinashe.Mutize@nt.gov.au](mailto:Tinashe.Mutize@nt.gov.au) |  |
| Tech staff (shipping) | TBA | TBA | TBA |
|  |  |  |  |

Territory Pathology Staff – Tennant Creek Hospital

| Title | Name | Email | Telephone |
| --- | --- | --- | --- |
| Lab Manager |  |  |  |
| Tech staff (shipping) | TBA | TBA | TBA |
|  |  |  |  |

**Figure 1: Flow chart of INFERR clinical Trial Laboratory sample collection, handling and processing**
