# Supplementary material for: INFERR-Iron infusion in haemodialysis study: INtravenous iron polymaltose for First Nations Australian patients with high FERRitin levels on haemodialysis—a protocol for a prospective open-label blinded endpoint randomised controlled trial
Source: Trials. 2021 Dec 2;22:868. doi: 10.1186/s13063-021-05854-w (PMC8641231; doi:10.1186/s13063-021-05854-w)
Supplement: Supplementary file 4 — Additional file 4. [file 13063_2021_5854_MOESM4_ESM.docx]

**Table 1: Target levels of markers of iron status in people with CKD from national and international guidelines**

| **Guidelines** | **Country/Region** | **Ferritin (ug/l)** | **TSAT (%)** |
| --- | --- | --- | --- |
| KDIGO | Worldwide | 100-500 | >20 |
| CARI | Australia & New Zealand | 200-500 | >20 |
| KDOQI | United States | 200-500 | 30-50 |
| UK Renal Association/NICE | United Kingdom | 250-500 | 30-50 |
| KDIGO: Kidney Disease: Improving Global Outcomes, CARI: Caring for Australasians with Renal insufficiency, KDOQI: Kidney Disease Outcome Quality Initiative, UK Renal Association/ NICE: United Kingdom Renal Association/ National Institute for Health and Clinical Excellence. | | | |
