# Supplementary material for: INFERR-Iron infusion in haemodialysis study: INtravenous iron polymaltose for First Nations Australian patients with high FERRitin levels on haemodialysis—a protocol for a prospective open-label blinded endpoint randomised controlled trial
Source: Trials. 2021 Dec 2;22:868. doi: 10.1186/s13063-021-05854-w (PMC8641231; doi:10.1186/s13063-021-05854-w)
Supplement: Supplementary file 5 — Additional file 5. [file 13063_2021_5854_MOESM5_ESM.docx]

**Table 2:** **Recently replaced protocol for iron infusion in use for haemodialysis patients in our units.**

| **Regimen** | **Iron Studies Result** | **IV Iron dose** |
| --- | --- | --- |
| A | TSAT <25 and Ferritin < 500 (Notify Dr before administering a 3rd consecutive treatment course) | Iron 200mg 3 times/week over 14 days (Total 6 doses) (repeat iron studies 2 weeks after 6th dose) |
| B | TSAT <25 and Ferritin <1000 | Iron 200mg weekly |
| C | TSAT 26- 50 and Ferritin <1000 | Iron 100mg weekly |
| D | TSAT < 50 and Ferritin < 1500 | Iron 50mg weekly |
| E | TSAT >50 and or Ferritin > 1500 with normal CRP | No replacement |
| F | TSAT > 50 and or Ferritin > 1500 with elevated CRP. | Withhold and repeat iron studies after 14 days without receiving iron |
| 1) Measure Iron Studies and CRP 3 monthly  2) Withhold intravenous Iron 14 days before Iron Studies and 14 days post blood transfusion  3) Ferritin > 1500 No iron replacement  Units of TSAT as a percentage, Ferritin in µg/L, elevated CRP>20mg/dl | | |
